# The Applicability of Current Turbidimetric Approaches for Analyzing Fibrin Fibers and Other Filamentous Networks

**DOI:** 10.3390/biom12060807

**Published:** 2022-06-09

**Authors:** Heather A. Belcher, Karen Litwa, Martin Guthold, Nathan E. Hudson

**Affiliations:** 1 Department of Physics, East Carolina University, Greenville, NC 27858, USA; belcherh17@students.ecu.edu; 2 Department of Anatomy & Cell Biology, East Carolina University, Greenville, NC 27858, USA; litwak16@ecu.edu; 3 Department of Physics, Wake Forest University, Winston-Salem, NC 27109, USA; gutholdm@wfu.edu

**Keywords:** turbidity, turbidimetry, fibrin, filamentous networks, light scattering

## Abstract

Turbidimetry is an experimental technique often used to study the structure of filamentous networks. To extract structural properties such as filament diameter from turbidimetric data, simplifications to light scattering theory must be employed. In this work, we evaluate the applicability of three commonly utilized turbidimetric analysis approaches, each using slightly different simplifications. We make a specific application towards analyzing fibrin fibers, which form the structural scaffold of blood clots, but the results are generalizable. Numerical simulations were utilized to assess the applicability of each approach across a range of fiber lengths and diameters. Simulation results indicated that all three turbidimetric approaches commonly underestimate fiber diameter, and that the “Carr-Hermans” approach, utilizing wavelengths in the range of 500–800 nm, provided <10% error for the largest number of diameter/length combinations. These theoretical results were confirmed, under select conditions, via the comparison of fiber diameters extracted from experimental turbidimetric data, with diameters obtained using super-resolution microscopy.

## 1. Introduction

Turbidity is defined as the fractional decrease in a primary beam’s intensity over a unit distance due to scattering as it passes through a sample [1]. Turbidity experiments are often performed at different wavelengths in a process referred to as turbidimetry [2]. With the assumption that the components of the network are randomly oriented, thin, cylindrical rods, light scattering theory can be used to extract parameters, such as the rod diameter and the mass–length ratio, from turbidimetry data. However, several different simplifications to light scattering theory have been employed to determine these parameters, leading to conflicting claims regarding the validity of these simplifications.

Turbidimetry analysis can and has been applied to many filamentous networks consisting of cylindrical components, such as collagen [3,4,5], nanocellulose particles (including cellulose nanofibers and cellulose nanocrystals) [6,7,8,9], and filamentous viruses [10]. In this work, we will specifically focus on its application in analyzing fibrin fibers, but our conclusions can be extended to other filamentous networks as well.

**Figure 1:**
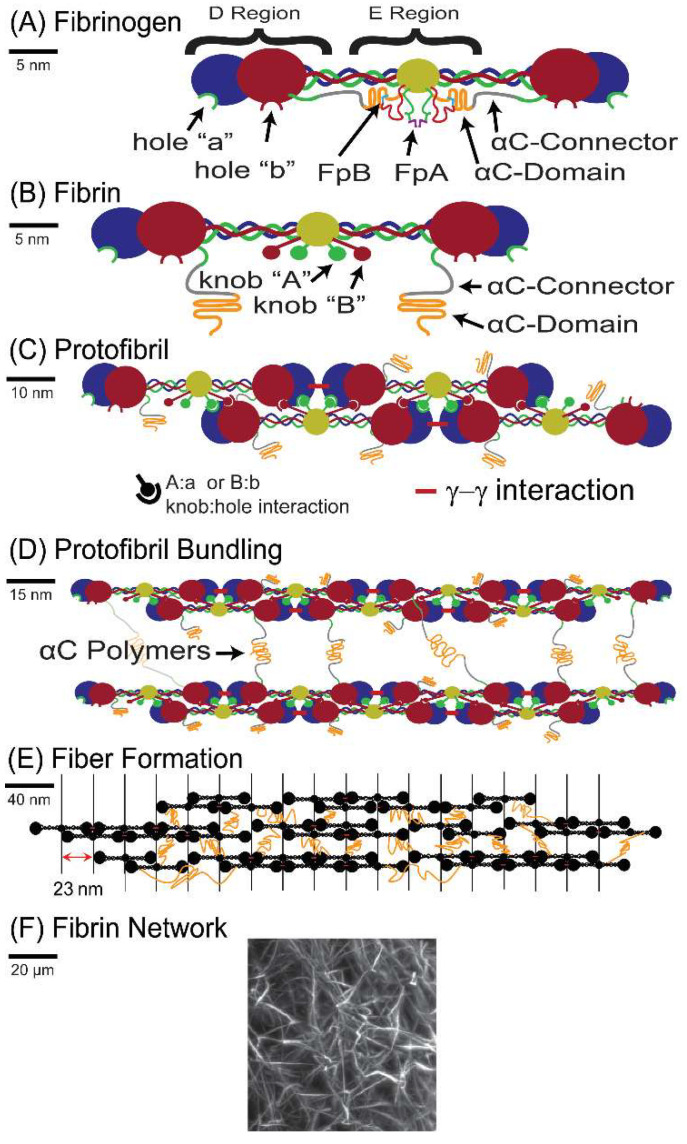
Schematic of the polymerization process: (**A**) Cartoon of the fibrinogen protein: the α chain is shown in green, β chain in red, and γ chain in blue; the disulfide bond-rich center of the molecule, where all six chains are connected, is depicted in yellow. (**B**) The fibrin molecule: upon thrombin cleavage of FpA and FpB, knob A and knob B are exposed to bind the respective hole a and hole b. (**C**) A half-staggered protofibril grows longitudinally as the knobs in the central region of one molecule bind to the holes in the distal region of two opposite molecules. (**D**) Lateral aggregation of protofibrils, likely mediated by interactions of the αC regions, which consist of the αC-connector and αC-domain. (**E**) Further aggregation of protofibrils into fibrin fibers. (**F**) A representative image of fibrin fibers in a gel. The image is a maximum intensity projection of a fibrin clot formed from human plasma spiked with 0.1% Alexa-488 labeled fibrinogen, imaged using a Bruker MuVi-SPIM light sheet microscope. Solid black lines on the left of each panel show the scale.

A network of fibrin fibers is the major structural component of blood clots, which form to stop the flow of blood in healthy hemostasis and pathological thrombosis. Fibrin clots consist of polymerized fibrin, which is the thrombin-activated form of the blood protein fibrinogen. Fibrinogen is a 340 kDa glycoprotein that has an elongated, trinodal structure with a length of 45 nm and a diameter of 2–5 nm (Figure 1A). Fibrinogen is made up of two sets of three polypeptide chains (Aα, Bβ, and γ chain) that are held together by 29 disulfide bonds [11]. The enzyme thrombin converts fibrinogen into fibrin by removing fibrinopeptides A and B (FpA and FpB), which is shown in Figure 1B. The fibrin molecules then polymerize into double-stranded protofibrils. Electron microscopy (EM) studies suggest the formation of half-staggered protofibrils as the center of one molecule binds to the ends of two other molecules, as shown in Figure 1C,D [11]. Recent light-scattering studies suggest that polymers may alternatively form from the binding of the center of one molecule to the end of another molecule in a “γ-ladder” [12]. In either case, once the protofibrils are formed, the exposure of knob ‘B’ leads to the aggregation of protofibrils into thicker fibers, likely mediated by interactions between the αC regions of the molecules [13] (Figure 1D,E). The formation of fibers continues to form a fibrin network (Figure 1F).

The structural properties of a fibrin network, such as pore size, and the structural properties of fibrin fibers, such as fiber length, diameter, mass–length ratio, and the number of protofibrils per cross-section, are controlled by many factors, including chemical and enzyme concentrations, blood flow rates, and cellular content (such as platelets and tissue-factor bearing cells) [14]. In addition, many pathological conditions, such as diabetes [15], myocardial infarction [16], ischemic stroke [17], and venous thromboembolism [18] result in gels and fibers with altered structural properties. To investigate these structural properties and how they are affected by environmental factors, it is important to develop and validate appropriate techniques for microstructure determination.

Turbidity is commonly used to study the kinetics of fibrin gel formation [19], where an increase in turbidity is interpreted as being caused by the bundling of protofibrils into fibers [20], and thus, increased light scattering is due to increased particle size. Turbidimetry, turbidity measurements at various wavelengths, is a commonly utilized approach in studying blood clot structure and function. Therefore, it is important to evaluate the effectiveness and validity of the different turbidimetric approaches for realistic fiber lengths, diameters, and densities.

The concept that turbidity changes with changing fiber size was first introduced by Ferry in the 1940s [21], and then further studied by Casassa in the 1950s [22] and by Marguerie and coworkers in the early 1970s [23]. Building on this work, Carr and Hermans developed a simplified light scattering theory to analyze turbidimetry data in the late 1970s, which is still commonly used today [24]. They confirmed the earlier works by Ferry, Casassa, and Marguerie et al. by replicating their experimental results, while also comparing the fiber properties obtained by turbidimetry to those obtained using the gel’s permeability [25]. They found that a simplified form of light scattering theory can provide reasonable values of the mass–length ratio, *μ*, and of the fiber diameter, *d*. Their simplified theory assumes a dilute solution of fibers and that the fibers are thin and long, meaning *d* << *λ* and *L* >> *λ*, where *L* is the fiber length and *λ* is the wavelength of the light (which was within a range of 350–650 nm in these studies). Moreover, they also assumed that the fibers were Rayleigh–Gans–Debye scatterers, meaning that the ratio between the optical index of the particles and the suspending medium is close to 1.

Building on the work of Carr and Hermans, Yeromonahos et al. [26] developed a modified theory for analyzing turbidimetry data. This method assumes that the fibers are Rayleigh–Gans–Debye scatterers, that the diameter is sufficiently small compared to the wavelength, that the length is sufficiently large compared to the wavelength, and that the concentration is sufficiently small so that the structure factor (explained in more detail later) can be neglected. However, the Yeromonahos approach uses different Taylor series expansions, which lead to different equations compared to those obtained by Carr and Hermans. The Yeromonahos approach is commonly used today for analyzing fibrin turbidimetry data [27,28,29].

In a subsequent letter to the editor, Yeromonahos et al. reported that in their original paper several equations were in error [30]. They proposed several modifications to their original equations; in particular, changing one term by a factor of 2/3, which has been used in some recent papers [2,19,31].

The method used by Carr and Hermans was evaluated by Ferri et al. [32] at specific fiber lengths (5, 10, 30, and 500 μm) and diameters (50, 100, 150, 200, and 300 nm). They pointed out that when the criterion that fibers are very thin and infinitely long is not met, as is often the case with fibrin, systematic errors in the fitting will be present. They also compared the Taylor series approximations used in the Carr–Hermans and Yeromonahos approaches, claiming the one used by Carr and Hermans leads to more accurate results. This claim was debated in a later paper [33]. Finally, they proposed a new framework for analyzing turbidimetry data by using a combination of experimental approaches to incorporate the fractal nature of the networks in the analysis.

In our work, we assess the accuracy of three commonly utilized methods (Carr–Hermans, original Yeromonahos, and corrected Yeromonahos) for analyzing turbidimetry data for fiber lengths (0.5–10 μm) and diameters (10–200 nm) that correspond to physiologically relevant values for fibrin gels. These ranges are based on findings by Ryan et al. [34] that fibers for conditions that are similar to those of blood (fibrinogen concentration of 3 mg/mL, pH of 7.5) contain fibrin fibers with average lengths of 0.3–4.8 μm and average diameters of 44–147 nm, as well as findings by Pretorius et al. [35] that fibrin fibers for healthy patients have average diameters of 89.97 ± 44.16 nm, while stroke patients have fibrin fibers with diameters of 27.37 ± 17.03 nm. We do not evaluate the approach suggested by Ferri et al. [32], since it requires a second experimental technique to determine network fractal properties, making it difficult to compare with the other approaches. We also investigate the effects of wavelength range and correcting for the wavelength dependence of the refractive index and specific refractive index increment on the accuracy of the turbidimetry results. We find that the validities of the approaches depend on the fiber lengths and diameters, as well as the wavelength ranges used for the measurements. We furthermore find that the approaches are far more accurate when the wavelength dependence of the refractive index and the specific refractive index increment are accounted for. Finally, at two fibrinogen concentrations, we compared the diameter values obtained from fitting the experimental turbidimetry data of a fibrin clot with each of the different turbidimetric approaches to diameter values obtained using super-resolution fluorescence microscopy.

## 2. Materials and Methods

### 2.1. Terminology

In reviewing papers discussing turbidity [2,24,26], we found inconsistencies in the language used, so we begin our methods section by clarifying the terminology, definitions, and applicable equations for determining turbidity with standard spectrophotometry instruments.

Although turbidity assumes that the only interaction with the light is scattering by the particulates, most spectrophotometers provide measurements in units of absorption or optical density. In reality, light can be either scattered or absorbed by a sample, leading to confusion when relating these processes to terms such as optical density. Therefore, it is important to understand how to convert data from common optical instruments to turbidity.

**Figure 2:**
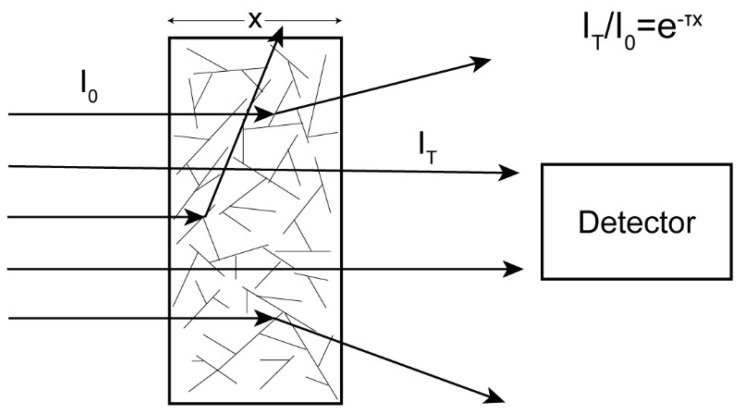
The incoming light enters the solution and is scattered by the rods. The transmitted light is collected by a detector. A ratio of the transmitted light to incoming light can be used to determine the turbidity.

Attenuance, *a*, is defined as the reduction in transmitted light through a sample due to absorption, scattering, or a combination of the two [36], and is given by:

(1) $$a=-\log T=-\log\left(\frac{I_{T}}{I_{0}}\right)$$

where T is the transmittance, defined as the ratio of transmitted light intensity, $I_{T}$, and incident light intensity, $I_{0}$. In the case of attenuation due to either absorption or scattering, the transmitted light intensity decreases exponentially with the thickness of the material through which the light passes [37]:

(2) $$\frac{I_{T}}{I_{0}}=10^{-\alpha x}$$

(3) $$\frac{I_{T}}{I_{0}}=e^{-\tau x}$$

where Equation (2) is for the case where all loss of light intensity is due to absorption and Equation (3) is for the case where all loss of light intensity is due to scattering, as illustrated in Figure 2. α is the absorption coefficient (in units of cm^−1^), τ is the turbidity (in units of cm^−1^), and x is the pathlength, or sample thickness (in units of cm).

The absorption coefficient, α, is then related to absorbance, A, by [36]:

(4) α=A/x

where the absorbance is defined as [36]:

(5) $$A=-\log\left(\frac{I_{T}}{I_{0}}\right)$$

which is the same as the definition of the attenuance in Equation (1), except that the definition of absorbance assumes that the only loss of light intensity is due to absorption, and not due to scattering, while for the attenuance, the loss of light intensity could be from either (or both).

The output quantity in spectrophotometry measurements is often given as absorbance, which assumes that all loss of light intensity is due to absorption. However, if both absorption and scattering are taking place, this quantity is actually the attenuance, and if scattering is the only interaction taking place, the quantity should be expressed in terms of turbidity. Thus, when studying turbidity using a spectrophotometer, it is often necessary to convert “absorption” readings into turbidity by setting Equation (2) equal to Equation (3), and setting the absorption coefficient in terms of absorbance:

(6) $$e^{-\tau x}=10^{-\left(\frac{A}{x}\right)x}=10^{-A}.$$

Taking the natural log of both sides and solving for turbidity provides the relationship between turbidity, τ, and the “absorbance” value, A, from a typical spectrophotometer:

(7) $$\tau =\frac{A}{x}\ln\left(10\right).$$

Several papers refer to turbidity in terms of optical density, OD [2,24,26], and so it is also necessary to understand how optical density relates to absorbance and turbidity. However, this is more complicated by the fact that the definition of optical density is inconsistent within the literature. Thus, the use of this optical density term is discouraged [36]. The most widely used definition of optical density in the literature is [24,38]:

(8) $$OD=-\log\left(\frac{I_{T}}{I_{0}}\right)$$

which makes optical density a unitless value with the same definition as absorbance, or attenuance if there is both scattering and absorption. This would mean that optical density and turbidity are related by:

(9) $$\tau =\frac{OD}{x}\ln\left(10\right).$$

However, in the original Yeromonahos paper [26], optical density was defined as:

(10) $$OD=-\frac{1}{x}\log\left(\frac{I_{T}}{I_{0}}\right)$$

which gives optical density units of cm^−1^. This led the authors to the incorrect relation that:

(11) $$\tau =1-e^{-OD\ln\left(10\right)}.$$

While this definition of turbidity was changed in a subsequent letter to the editor [30] to τ=OD ln(10), the incorrect definition (Equation (11)) has been repeated in more recent papers [19,27].

The lack of the pathlength factor in the updated Yeromonahos definition of turbidity has caused confusion in and of itself, as it has resulted in some researchers inserting the pathlength into the equation incorrectly [19,27]. While the definition of turbidity as τ=OD ln(10) using optical density with units of cm^−1^ makes sense when using a pathlength of 1 cm, such as with a standard 1 cm wide cuvette, it may result in errors when using a pathlength other than 1 cm (such as in a 96-well plate). In those cases, it is necessary to calculate the pathlength. This can be performed by loading reference wells with the same volume of water as the liquid volume in the sample wells and taking absorbance measurements at ~975 nm (the absorption peak of water) and 900 nm (a reference wavelength), and then using the equation:

(12) $$x=\frac{A_{\left(975\;nm\right)}-A_{\left(900\;nm\right)}}{K-factor}$$

where the K-factor is a measure of the absorbance of water at the absorption peak minus the absorbance of water at the reference wavelength for a pathlength of 1 cm [39]. The pathlength can also be determined via a measurement of the height of the sample within the well, although this is difficult to obtain accurately, due to the meniscus of the sample surface.

### 2.2. Light Scattering Using Finite Length Cylinders

The Rayleigh ratio is a quantity that is used to characterize the scattering intensity at the scattering angle *θ* [40]. According to light scattering theory, the Rayleigh ratio for thin, dilute, randomly oriented, cylindrical fibers is [32,38]:

(13) R(q)=KcMP(q,L,d)S(q)

where $q=\left(\frac{4\pi }{\lambda }\right)sin\left(\frac{\theta }{2}\right)$ is the scattering wavevector, $K=\left(\frac{4\pi^{2}}{N_{A}\lambda^{4}}\right)n^{2}{\left(\frac{dn}{dc}\right)}^{2}$ is the optical constant for vertically polarized incident light, where $N_{A}$ is Avogadro’s number, λ is the wavelength, n is the refractive index of the solution, and dn/dc is the specific refractive index increment of the solute in the solvent. c is the concentration of fibrinogen. $M=N_{A}\left(\frac{\pi }{4}\right)\rho d^{2}L=\mu L$ is the molecular weight of a cylindrical fiber, where ρ is the density, d is the diameter, L is the length, and μ is the mass–length ratio (the molecular weight per unit length of the fiber). Assuming that the fibers are Rayleigh–Gans–Debye scatterers (the relative refractive index of the fibers is close to that of the surrounding medium), $P\left(q,L,d\right)=P_{rod}\left(q,L\right)S_{sec}\left(q,d\right)$ is the form factor. $P_{rod}\left(q,L\right)=\frac{2Si\left(qL\right)}{qL}-{\left[\frac{\sin\left(\frac{qL}{2}\right)}{\frac{qL}{2}}\right]}^{2}$ is the form factor of a thin rod, where the sine integral, $Si\left(qL\right)=\int_{0}^{qL}\frac{sinx}{x}dx$, and $S_{sec}\left(q,d\right)={\left[\frac{2J_{1}\left(\frac{qd}{2}\right)}{\frac{qd}{2}}\right]}^{2}$ is the cross section of a thin rod. S(q) is the structure factor, which is assumed to be 1, meaning that the fibers are non-interacting with each other.

The turbidity is equivalent to the Rayleigh ratio integrated over the entire solid angle *dΩ*:

(14) $$\tau =\int R\left(\theta \right)\frac{1+\cos^{2}\theta }{2}d\Omega$$

and since the turbidity is a function of the Rayleigh ratio, which is a function of the diameter and mass–length ratio, the turbidity can be used to obtain the diameter and the mass–length ratio.

It should be noted that our derivations assume the incoming light is vertically polarized, following the approach used by Ferri et al. [32]; however, Carr and Hermans [24] use unpolarized light, as seen by their differing definitions of the optical constant, and arrive at the same final equations.

### 2.3. Carr–Hermans Approach

Because Equation (14) cannot be solved analytically, in order to apply it to experimental data, simplifications must be made to extract the fiber properties from the turbidimetry data. Perhaps the best-known approach to determining the diameter and mass–length ratio from the turbidity is that described by Carr and Hermans [24]. Carr and Hermans use Equations (13) and (14) for their basis; however, they make the assumption that $\frac{qd}{2}\ll 1$ by assuming that the diameter is much less than the wavelength. By Taylor expanding the Bessel function and keeping only the first two terms, $S_{sec}$ becomes:

(15) $$\;S_{sec}=1-\frac{{\left(qd\right)}^{2}}{16}+\frac{{\left(qd\right)}^{4}}{1024}.$$

Then with the assumption that $\frac{qd}{2}\ll 1$, the last term can be neglected, leading to the approximation that $S_{sec}\approx 1-{\left(\frac{qd}{4}\right)}^{2}.$ They also make the assumption that the fibers are infinitely long, which leads them to simplify $Si\left(qL\right)\approx \frac{\pi }{2}$ and ${\left[\frac{\sin\left(\frac{qL}{2}\right)}{\frac{qL}{2}}\right]}^{2}\approx 0$, allowing them to make the approximation that $P_{rod}\approx \frac{\pi }{qL}$. Plugging the new value for the Rayleigh ratio into Equation (14) and integrating then gives:

(16) $$\tau =\frac{88}{15}\frac{\pi^{3}}{N_{A}}n{\left(\frac{dn}{dc}\right)}^{2}c\frac{1}{\lambda^{3}}\mu \left[1-\frac{23}{77}\pi^{2}n^{2}d^{2}\frac{1}{\lambda^{2}}\right].$$

Carr and Hermans then use the Taylor expansion $\frac{1}{x}=\overset{\infty }{\underset{n=0}{\sum }}{\left(-1\right)}^{n}{\left(x-1\right)}^{n}$ where $x=1-\frac{23}{77}\pi^{2}n^{2}d^{2}\frac{1}{\lambda^{2}}$, and they make the assumption that $\frac{23}{77}\pi^{2}n^{2}d^{2}\frac{1}{\lambda^{2}}\ll 1$ by assuming the diameter is much smaller than the wavelength, so that they can use the first two terms of the Taylor expansion to make the approximation that ${\left[1-\frac{23}{77}\pi^{2}n^{2}d^{2}\frac{1}{\lambda^{2}}\right]}^{-1}=1+\frac{23}{77}\pi^{2}n^{2}d^{2}\frac{1}{\lambda^{2}}$. This leads to the equation:

(17) $$\frac{c}{\tau \lambda^{3}}=\frac{N_{A}}{\left(\frac{88}{15}\right)\pi^{3}n{\left(\frac{dn}{dc}\right)}^{2}\mu }\left[1+\frac{23}{77}\pi^{2}n^{2}d^{2}\frac{1}{\lambda^{2}}\right].$$

With this equation, for a plot with $\frac{c}{\tau \lambda^{3}}$ as the *y*-axis and $\frac{1}{\lambda^{2}}$ as the *x*-axis, the y-intercept and slope can be used to solve for the mass–length ratio, and this can then be used along with the slope to solve for the diameter.

The validities of the different assumptions are discussed in Section S.1 of the Supporting Information.

### 2.4. Original Yeromonahos Approach

The original approach described by Yeromonahos et al. [26] is very similar to that of Carr and Hermans, except that they do not make the assumption that $\frac{23}{77}\pi^{2}n^{2}d^{2}\frac{1}{\lambda^{2}}\ll 1$, and so they use Equation (16) as their final equation. Rewriting this equation, it becomes:

(18) $$\tau \lambda^{5}=\frac{1}{N_{A}}2\pi^{3}cn\mu {\left(\frac{dn}{dc}\right)}^{2}\left(\frac{44}{15}\right)\left[\lambda^{2}-\frac{184}{154}\pi^{2}n^{2}r^{2}\right]$$

although the factor $\frac{1}{N_{A}}$ was missing in the equation, as given in the Yeromonahos paper. Then, for a plot of $\tau \lambda^{5}$ versus $\lambda^{2}$, the slope can be used to solve for the mass–length ratio and then the y-intercept and slope can be used to solve for the radius, or in turn, the diameter.

Although Yeromonahos later wrote a Letter to the Editor attempting to correct this equation [30], we will still evaluate Equation (18) because it is still commonly used within the fibrinogen community [20,27,28,29]. This equation will be referred to as the “original Yeromonahos” approach, and the corrected version will be referred to as the “corrected Yeromonahos” approach.

### 2.5. Corrected Yeromonahos Approach

The corrected Yeromonahos approach changes the 184/154 term of the original equation (Equation (18)) by a factor of 2/3, leading to the final fitting equation:

(19) $$\tau \lambda^{5}=\frac{1}{N_{A}}2\pi^{3}cn\mu {\left(\frac{dn}{dc}\right)}^{2}\left(\frac{44}{15}\right)\left[\lambda^{2}-\frac{184}{231}\pi^{2}n^{2}r^{2}\right].$$

Then, using a plot of $\tau \lambda^{5}$ versus $\lambda^{2}$, the slope and y-intercept can be used to extract the diameter and the mass–length ratio.

The change in the 184/154 term is described as being due to averaging the form factor, P(q,L,d), over all solid angles [30]; however, it was done so erroneously, as discussed in more detail in Section S.2 of the Supporting Information. Thus, the “corrected” equation still has flaws.

### 2.6. Data Generation and Curve Fitting

**Figure 3:**
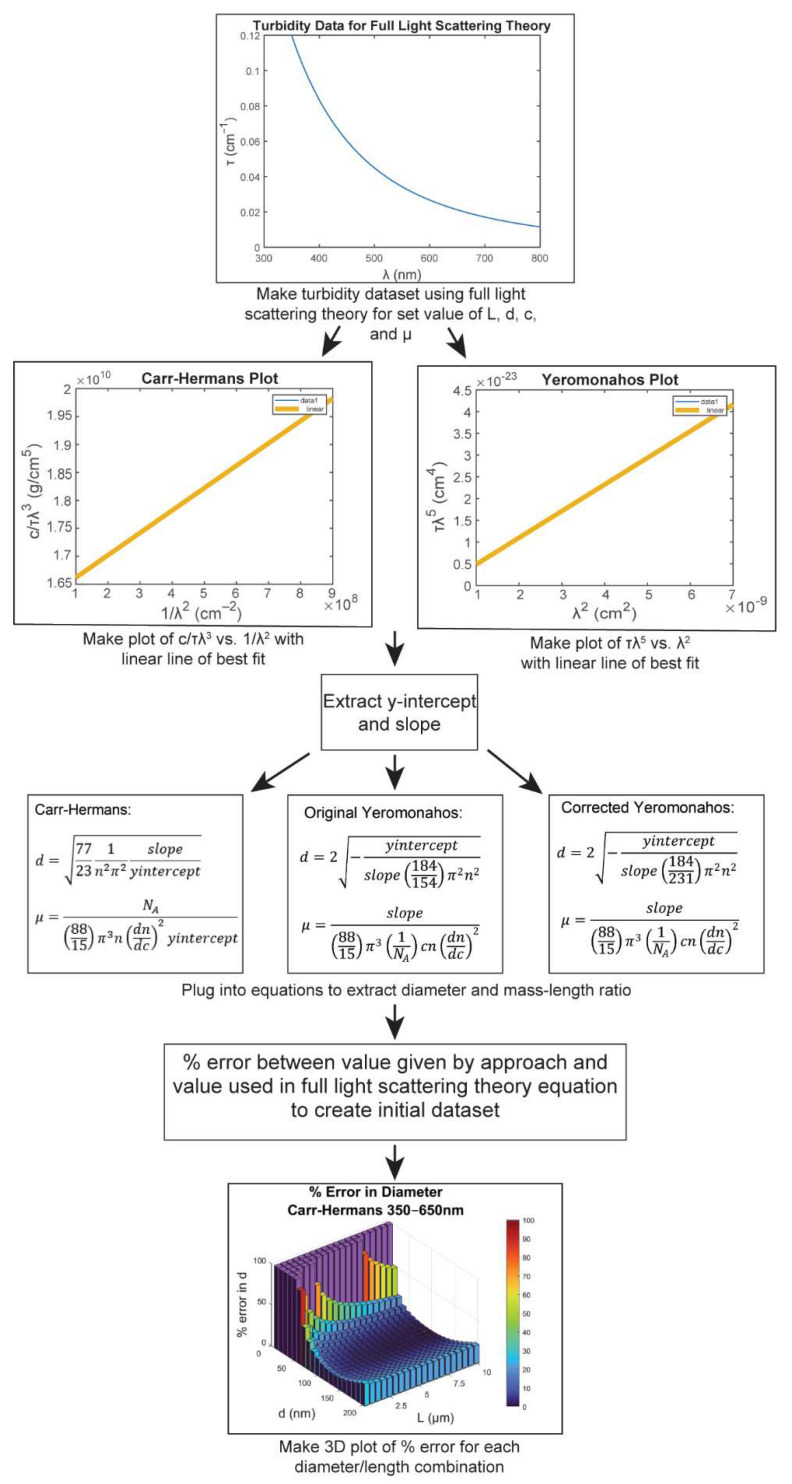
An outline of the methods for determining the percent error in each approach, using constant values of n and dn/dc.

Data were numerically generated using a custom code in Mathematica (Wolfram, Champaign, IL). The data generation and analysis pipeline is outlined in Figure 3. Numerical integration was conducted to create a turbidity dataset using full light scattering theory (Equations (13) and (14)) for fixed values of length, diameter, concentration, and mass–length ratio. The fibrinogen concentration (0.0001 g/cm^3^) and mass–length ratio (4.73 × 10^12^ Da/cm) were chosen to match the values used in Ferri et al. [32]; however, the percent error is independent of the concentration and the mass–length ratio, as described in Section S.6 of the Supporting Information. To confirm that the Mathematica numerical integration was correct, we also performed a trapezoidal rule numerical summation using a custom code, which generated similar results. With data from the full light scattering theory in hand, datasets for either $\frac{c}{\tau \lambda^{3}}$ versus $\frac{1}{\lambda^{2}}$ (Carr–Hermans) or $\tau \lambda^{5}$ versus $\lambda^{2}$ (Yeromonahos) were created (or the wavelength-corrected formats discussed in Section S.3 in the Supporting Information). Using a custom Mathematica code, the data were then fit with the Carr–Hermans, the original Yeromonahos, or the corrected Yeromonahos equations (Equations (17)–(19), respectively). The diameter and mass–length ratio obtained from the linear fits, and the percent error between the value obtained from the fits and the value used to create the dataset using full light scattering theory was determined. A parameter sweep was then used to calculate the percent error for the physiologically relevant fiber length and diameter values, which was then plotted in a 3D heatmap using a “bar3” command in MATLAB (The Mathworks Inc., Natick, MA, USA).

The three approaches were investigated for the wavelength range of 350–650 nm, which is the wavelength range used in the Carr–Hermans paper [24], as well as the wavelength range of 500–800 nm, which is the wavelength range used by Yeromonahos [26]. Carr and Hermans used the wavelength range of 350–650 nm because it is the most linear portion of the $\frac{c}{\tau \lambda^{3}}$ versus $\frac{1}{\lambda^{2}}$ plots, as can be seen in Figure S4 in the Supporting Information. Although Yeromonahos does not explain the reasoning for using a wavelength range of 500–800 nm, it was argued by García et al. [33] that by using this wavelength range, it is not as necessary to correct for the wavelength dependence of n and dn/dc, as it results in less than a 5% change in the data. We investigate this claim further in the Supporting Information Section S.3.

### 2.7. Experimental Turbidimetry Analysis

Each of the turbidimetric approaches were applied to experimentally obtained data from fibrin clots formed under two different conditions. One set of experiments formed a clot using final concentrations of 0.5 mg/mL peak 1 fibrinogen (Enzyme Research Labs, Indianapolis, IN, USA) and 0.1 NIH-U/mL human alpha thrombin (Enzyme Research Labs, Indianapolis, IN, USA). The other set of experiments formed a clot using final concentrations of 1 mg/mL peak 1 fibrinogen, 0.1 NIH-U/mL human alpha thrombin, and 25 L-U/mL FXIIIa. In both sets of experiments, polymerization occurred at 37 °C in a buffer containing 150 mM sodium chloride, 20 mM HEPES, and 5 mM calcium chloride, pH 7.4, in order to determine the fiber diameter. “Absorbance” readings were taken after one hour of polymerization at 37 °C in a masked 1 cm wide cuvette using a Thermo Scientific NanoDrop 2000c spectrophotometer, with measurements taken every 10 nm over a range of 350–800 nm. Fitting of the data was conducted using each of the turbidimetric approaches on the data ranging over 350–650 nm, as well as 500–800 nm. This was performed with the assumption of constant values of n and dn/dc, as well as with the wavelength dependence of those terms being accounted for. The equations for calculating the diameter, as well as the uncertainty in the diameter, are shown in Section S.8 of the Supporting Information.

### 2.8. Stochastic Optical Reconstruction Microscopy (STORM) Imaging

STORM imaging was performed on fibrin clots formed from final concentrations of 0.5 mg/mL peak 1 fibrinogen, 0.1 NIH-U/mL human alpha thrombin, and 0.0075 mg/mL AlexaFluor-647-labeled fibrinogen, as well as a clot formed from final concentrations of 1 mg/mL peak 1 fibrinogen, 0.1 NIH-U/mL human alpha thrombin, 25 L-U/mL FXIIIa, and 0.015 mg/mL AlexaFluor-647-labeled fibrinogen, both in HEPES buffered saline (HBS; 150 mM NaCl, and 20 mM HEPES, pH 7.4) with 5 mM calcium chloride. To prepare the samples, 5 μL fibrinogen solution (containing the wild-type fibrinogen, AlexaFluor-647-labeled fibrinogen, and FXIIIa if present) was spread out onto a cover glass and combined with 5 μL thrombin solution. Samples were placed into a closed Petri dish with a damp Kimwipe to create a humidified container, and were allowed to polymerize for one hour at 37 °C. Following polymerization, the samples were topped with 5 μL HBS buffer and 5 μL Vectashield mounting medium to decrease photobleaching of the fluorophores. The cover glass containing the sample was then carefully placed onto a microscope slide, with the fibrin clot between the two, and sealed around the edges using fingernail polish.

Stochastic optical reconstruction microscopy (STORM) images were acquired using Nikon NIS-Elements AR software version 5.11.00 and an Apo TIRF 100× objective (1.49 NA) on a Nikon Eclipse Ti2 inverted microscope, equipped with 405 and 640 nm lasers. Imaging was performed in a slice where fibers were primarily oriented in the imaging plane to ensure accurate fiber diameter measurements. Images were acquired at an image size of 256 × 256 pixels for 20,000 frames using a ProEM HS EMCCD camera (Princeton Instruments), 16-bit with no binning. EM gain was set to 20 MHz with an EM gain multiplier of 300 and a conversion gain multiplier of 1. The 640 nm laser power was set to 100%, and the “Adjusted Laser Powers” setting provided by the NIS-Elements software was used to control the photoswitching of the AlexaFluor-647 fluorophores through activation using the 405 nm laser and excitation using the 640 nm laser.

STORM images were reconstructed from the acquired raw .nd2 files using the NIS-Elements software with the following settings: “Auto Fit ROI”, minimum height 1000, maximum height 65,535; CCD Baseline was set to 100, minimum width 200 nm, maximum width 700 nm, initial fit width 300 nm, maximum axial ratio 2.5, and a maximum displacement of 1 pixel. At the end of the run, the files were processed into .bin files, which contained the molecule count information with the coordinates and the intensity of each blinking event. Drift correction was performed using the automatic drift correction option in the NIS-Elements software. After processing, the final STORM images were displayed as a collection of Gaussian spots representing each blinking event. A 30 × 30 pixel region of interest was captured for each fiber within the image, and saved as a .nd2 file for further analysis. Subsequent analysis of the processed .nd2 files was performed with ImageJ (http://rsbweb.nih.gov/ij/ accessed on 23 March 2020) software using the line segment measuring tool to obtain the fiber diameter. This was performed for two samples at a 0.5 mg/mL fibrinogen concentration, with a total of 77 fiber diameters measured, and one sample at the 1 mg/mL fibrinogen concentration, with a total of 128 fiber diameters measured.

## 3. Results

### 3.1. Importance of Including the Wavelength Dependence of n and dn/dc in Analyzing Numerically Generated Turbidimetric Data

Many studies using turbidimetry to determine the diameter and mass–length ratio assume constant values of n and dn/dc [2,24,26]; however, these values actually vary with wavelength. The wavelength dependence can be accounted for by using the Cauchy relations [32,41]:

(20) $$n\left(\lambda \right)=A_{1}+\frac{B_{1}}{\lambda^{2}}$$

(21) $$\frac{dn}{dc}\left(\lambda \right)=A_{2}+\frac{B_{2}}{\lambda^{2}}$$

where for a fibrin gel in HBS buffer (150 mM sodium chloride and 20 mM HEPES, pH 7.4), $A_{1}=1.3264$, $B_{1}=3129.8$, $A_{2}=0.1859$, and $B_{2}=1640$ (the software SEDNTERP [42] was used to determine the equation for n, and SEDFIT [43,44] was used to determine the equation for dn/dc), where λ is in nm.

**Figure 4:**
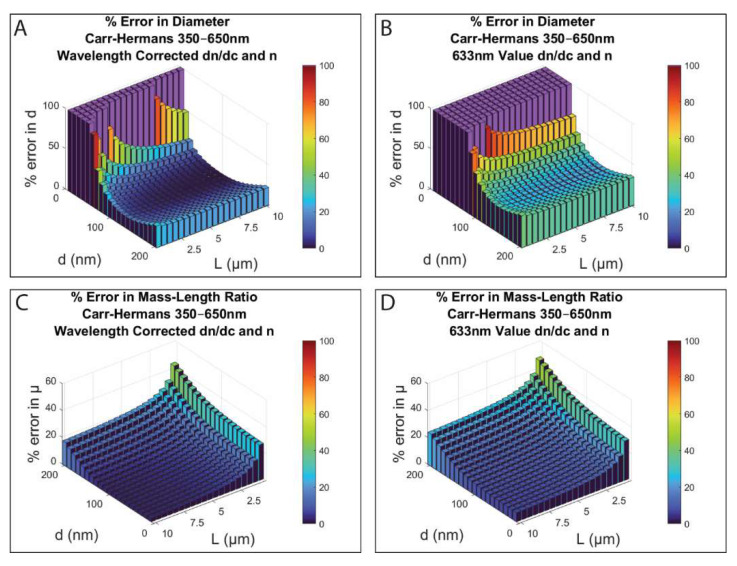
Percent error in diameter (**A**,**B**) and in mass–length ratio (**C**,**D**) between the values obtained from fitting the Carr–Hermans approach to the full light scattering theory dataset and the values used to create the initial dataset for lengths of 0.5–10 μm and diameters of 10–200 nm. In (**A**,**C**), the wavelength dependence of n and dn/dc was accounted for (Equations (20) and (21); in (**B**,**D**), constant values for n and dn/dc at 633 nm were used. (c = 0.0001 g/cm^3^, μ = 4.73 × 10^12^ Da/cm, HBS buffer; the purple bars represent imaginary diameter values calculated from the fit and were assigned a value of 100% error for plotting purposes; note that (**A**,**B**) have a different viewing angle to (**C**,**D**).

As can be seen in Figure 4, using a constant value of n and dn/dc, instead of accounting for the wavelength dependence of these quantities, results in a significantly increased error in the extracted diameter and the mass–length ratio values. This is particularly apparent for smaller diameter values, and especially in the calculation of the diameter (Carr–Hermans approach for a 350–650 nm wavelength range shown). The amount of error added for all three approaches when using the constant value at 633 nm compared to the values when accounting for the wavelength dependence can be seen in Figures S1 and S2 in the Supporting Information for both the wavelength ranges investigated.

It has also been found by others for experimental data [45] that using a constant value for n and dn/dc, rather than accounting for the wavelength dependence of those terms, can lead to an average difference of 24% in the calculated fiber radius and 3% in the calculated mass–length ratio. This is for fibers with diameters ranging from 110.4 to 218.8 nm, based on their calculations. Although they used a different wavelength range (450–900 nm), their length values are not given, and a different buffer was used, this appears to be in good agreement with our results. A summary of the average difference in the percent error values when using a constant n and dn/dc compared to using wavelength corrected n and dn/dc is provided in Table 1 for each of the different approaches and wavelength ranges.

Due to the increased error when using a constant value for n and dn/dc, for all subsequent results the full light scattering theory equation was corrected for wavelength dependence when creating the initial theoretical turbidity dataset, and the approaches were also corrected for wavelength dependence when being fit to the dataset, as shown in the methods outlined in Figure S5 in the Supporting Information.

When accounting for the wavelength dependence of n and dn/dc, it is necessary to rewrite the equations and to adjust the plot format in order to remove the n(λ) and dn/dc(λ) factors from the calculations of diameter and mass–length ratio, since they are now dependent on the wavelength. The way in which this is performed is described in Section S.3 in the Supporting Information.

### 3.2. Numerical Simulation Estimates of Diameter

**Figure 5:**
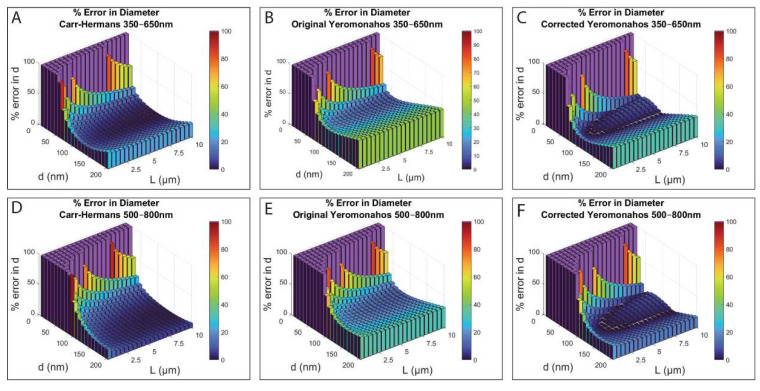
Percent error between the diameter obtained from fitting the three approaches to theoretical turbidity values created using full light scattering theory, and the value used to create the initial dataset for lengths of 0.5–10 μm and diameters of 10–200 nm for wavelength ranges of 350–650 nm (**A**–**C**) and 500–800 nm (**D**–**F**). (c = 0.0001 g/cm^3^, μ = 4.73 × 10^12^ Da/cm, dn/dc and n corrected for wavelength dependence for fibers in HBS buffer; the purple bars represent imaginary diameter values calculated from the fit, and were assigned a value of 100% error for plotting purposes).

Because the three approaches are routinely used to extract gel structural properties from light scattering data, we wanted to evaluate the amount of error in the diameter and the mass–length ratio values obtained from the different data-fitting approaches. The percent error in the values of the diameter can be seen in Figure 5, when the data from either the 350–650 nm wavelength range (used by Carr and Hermans [24]) or the 500–800 nm wavelength range (used by Yeromonahos [26]) were used.

As can be seen in Figure 5, all three approaches result in imaginary diameter values for thin fibers (diameter less of than ~50 nm) for both wavelength ranges, shown by the purple bars and assigned a percent error value of 100% for plotting purposes. For thicker fibers (diameters over a range of 150–200 nm), all three approaches show better fits with the 500–800 nm wavelength range. The Carr–Hermans approach and the corrected Yeromonahos approach for the two wavelength ranges are best for fibers of intermediate thickness (diameters over a range of 50–150 nm), and the Carr–Hermans approach with a wavelength range of 500–800 nm is best for thicker fibers (with diameters of 150–200 nm), although the error does increase considerably for diameters above 200 nm, as shown in Section S.7 of the Supporting Information. Some diameter values, obtained with the three approaches across this range of diameters and lengths, are shown in Section S.4 in the Supporting Information. The Carr–Hermans approach and the original Yeromonahos approach always result in underestimated values of the diameter when compared to the values used to create the dataset using full light scattering theory for all of the diameter/length combinations investigated. The corrected Yeromonahos approach results in a mostly underestimated diameter value, except for the area of increased error at intermediate diameters (~50–150 nm) seen in Figure 5, in which the corrected Yeromonahos approach overestimates the diameter values. Because the percent error plotted in Figure 5 involves taking the absolute value of the difference in the diameter value used to create the initial dataset using full light scattering theory and the calculated value obtained from the fit, the transition from under- to overestimating the diameter results in the “saddle” feature seen in panels C and F.

A summary of which approach is best for each diameter/length combination, and which approaches result in a less than 10 percent error, can be seen in Figure S6 in the Supporting Information.

### 3.3. Numerical Simulation Estimates of Mass–Length Ratio

**Figure 6:**
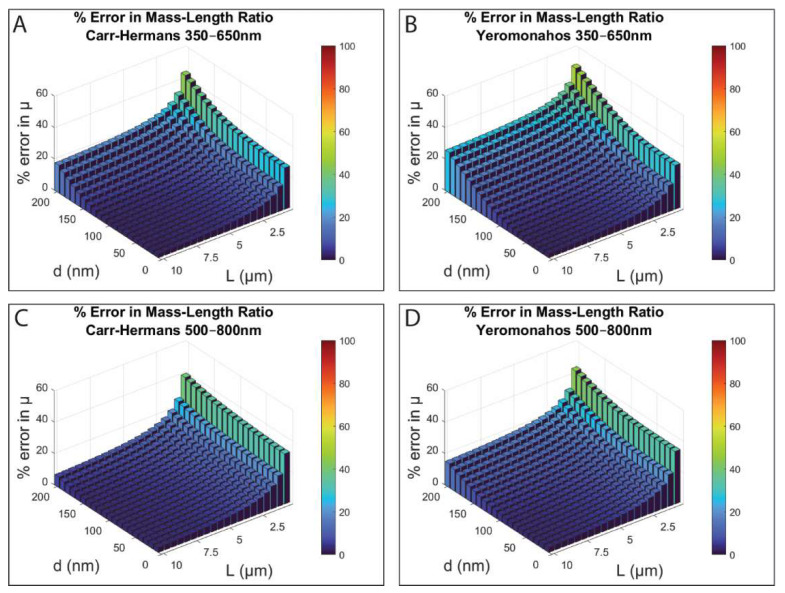
Percent error between the mass–length ratio obtained from fitting the approaches to theoretical turbidity values created using full light scattering theory and the value used to create the initial dataset for lengths of 0.5–10 μm and diameters of 10–200 nm for wavelength ranges of 350–650 nm (**A**,**B**) and 500–800 nm (**C**,**D**). (c = 0.0001 g/cm^3^, μ = 4.73 × 10^12^ Da/cm, dn/dc and n corrected for wavelength dependence for fibers in HBS buffer).

The percent error in the mass–length ratio obtained from the Carr–Hermans approach (Equation (17)) and the original and corrected Yeromonahos approaches (Equations (18) and (19), respectively) fit to the full light scattering theory data compared to the value used to create the dataset are shown in Figure 6 for the two wavelength ranges. Note that the viewing angle is rotated 180° from what it was for the diameter error plots in Figure 5, and the percent error is the same for both the original and the corrected Yeromonahos approaches, since the change in the term does not have an impact on the calculation of the mass–length ratio.

Overall, there is much less error in the values of the mass–length ratio than with the diameter values. However, both wavelength ranges for all three of the approaches show an increased amount of error in the values of the mass–length ratio when the fibers are short (less than ~2 μm). All three of the approaches and both wavelength ranges appear to provide the most accurate values of the mass–length ratio when the fibers are thin (less than ~100 nm diameter) and long (5–10 μm length) and the Carr–Hermans approach from 500–800 nm also appears to provide accurate values when the fibers are thick (with diameters in the range of 100–200 nm), although the error does increase considerably for diameters above 200 nm, as shown in Section S.7 of the Supporting Information. Some actual values of the mass–length ratio given by the approaches across this range of diameters and lengths are shown in Section S.5 in the Supporting Information. The Carr–Hermans approach and the Yeromonahos approaches all result in underestimations of the mass–length ratio values compared to the values used to create the dataset when using full light-scattering theory for all diameter/length combinations investigated.

A summary of which approach is best for each diameter/length combination, and which approaches result in a less than 10 percent error, can be seen in Figure S7 in the Supporting Information. It shows that although the Carr–Hermans approach and the Yeromonahos approach for both wavelength ranges provide less than 10% error in the mass–length ratios for most diameter/length combinations, the Carr–Hermans approach from 350–650 nm is best when the fibers have diameters of less than ~140 nm, and the Carr–Hermans approach from 500–800 nm is best when the fibers have diameters of 140–200 nm.

### 3.4. Experimentally Obtained Diameter Values

Each of the turbidimetric approaches were fit to the experimental data obtained from either a fibrin clot formed from 0.5 mg/mL fibrinogen and 0.1 NIH-U/mL thrombin, or a fibrin clot formed from 1 mg/mL fibrinogen, 0.1 NIH-U/mL, and 25 L-U/mL FXIIIa, in order to determine fiber diameter. These fibrinogen concentrations were chosen in order for the clot to be dilute enough so that multiple scattering should be negligible [33], and to provide experimental analysis for two different clot conditions, which should provide different fiber thicknesses [34]. Data was analyzed for a wavelength range of 350–650 nm, as well as 500–800 nm. It was analyzed using constant values for n and dn/dc, as well as accounting for their wavelength dependence. For the constant values of n and dn/dc, the spectrally averaged values were used.

The diameter values and uncertainty obtained from fitting the data with each of the turbidimetric approaches for both clot conditions are shown in Table 2. For the clot formed from 0.5 mg/mL fibrinogen, the plots of the experimental data are shown in Figures S12 and S13 in the Supporting Information. For the clot formed from 1 mg/mL fibrinogen, the plots of the experimental data are shown in Figures S14 and S15. The equations for determining the uncertainty in the diameter for each of the approaches are shown in Equations (S.22), (S.24), (S.26) and (S.28) of the Supporting Information.

For comparison, the fibers of the same fibrinogen and thrombin concentrations were also imaged using a Nikon STORM (Stochastic Optical Reconstruction Microscopy) system. Typical visible light microscopes have a resolution limit of ~250 nm, with anything below this appearing as a diffraction-limited blur [46]. Since fiber diameters can vary over a range of 16–391 nm [34], some fibers would fall below the resolution limit, making accurate diameter estimates impossible. However, STORM microscopy is able to obtain over 10× improved resolution through the use of optically switchable fluorophores that can be switched between a nonfluorescent and a fluorescent state by exposure to light [47]. By using optically switchable fluorophores, only a small number of fluorophores are excited at a time, allowing for a high degree of accuracy in the localization of individual fluorophores with Gaussian fitting [48]. This allows for an imaging resolution of approximately 20 nm [47]. The smallest diameter that we recorded was 99 nm (see results), which is well above this resolution limit.

**Figure 7:**
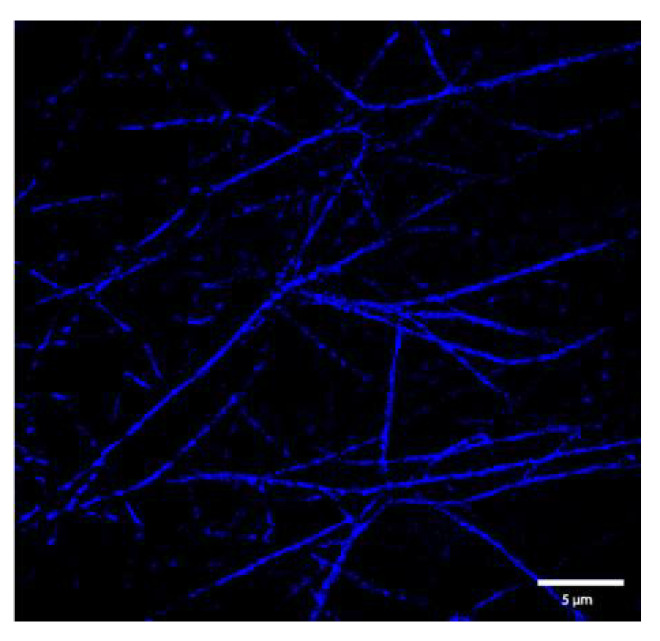
Stochastic optical reconstruction microscopy (STORM) image of a clot formed from 0.5 mg/mL fibrinogen and 0.1 NIH-U/mL thrombin (in HBS buffer + 5 mM calcium chloride), with AlexaFluor-647 labeled fibrinogen added at a concentration of 1/65 that of the wild-type fibrinogen to allow for fluorescence imaging, obtained on a Nikon Ti2-E inverted microscope using a 100× oil objective.

One of the STORM images obtained is shown in Figure 7. The average diameter value and standard deviation of the fibers obtained using STORM imaging for both clot conditions are shown in Table 2. A histogram of all the diameter values obtained is provided in Figure S16 in the Supporting Information for the clot formed from 0.5 mg/mL fibrinogen, and in Figure S17 for the clot formed from 1 mg/mL fibrinogen.

## 4. Discussion

There is an interest in determining the structural parameters of fibrin fibers, because these parameters provide fundamental information regarding fibrin clot formation, and they have been correlated to diseases. Light scattering theory provides a connection between microscopic clot parameters (namely fiber diameter and mass–length ratio), and turbidity, which can be measured easily. However, extraction of the fiber diameter and mass–length ratio from turbidity data requires approximations to light scattering theory so that the scattering equations can be fit to turbidity versus wavelength data. We evaluated the validity of the three most commonly used approximations for physiologically relevant fiber lengths and diameters: the Carr–Hermans, the original Yeromonahos, and the corrected Yeromonahos approach, by applying them to turbidity versus wavelength datasets that were created from full light scattering theory. We then evaluated these theoretical results by applying the turbidimetric approaches to experimentally obtained data from a fibrin clot and comparing the obtained diameter values to those obtained from super-resolution fluorescence imaging.

### 4.1. Theoretical Analysis

Based on the numerical simulations, we found that while the three different approaches worked reasonably well for some fiber diameters and lengths, they also produced unacceptably large errors or imaginary values for other fiber diameters and lengths. Thus, caution needs to be used when extracting fibrin fiber structural properties from turbidity versus wavelength data.

The imaginary results for the diameters for thin fibers (less than ~50 nm) with the three approaches are explained by Figure S8 in the Supporting Information. As can be seen with the Carr–Hermans plots, for the small diameter values (10 and 50 nm), the slopes of the plots are negative, whereas they are positive for diameters of 100, 150, and 200 nm. Since the diameter is solved for by taking the square root of the slope, this translates to an imaginary value of the diameter. Then, for the Yeromonahos plots, there is a positive y-intercept for fibers with small diameters, and since the diameter is solved for by taking the square root of the negative of the y-intercept, a positive y-intercept would result in an imaginary value of the diameter. The imaginary numbers were assigned a value of 100% error for plotting purposes, and they are shown in purple in Figure 5. The reasoning for this change in slope and y-intercept for the full light scattering theory data is unclear, due to the fact that Equation (14) cannot be integrated analytically, and therefore, it cannot be written in terms of the plotted y’ and x’ variables used for the fitting approaches ($\frac{{\left[\frac{dn}{dc}\left(\lambda \right)\right]}^{2}}{{<\frac{dn}{dc}>}^{2}}\frac{n\left(\lambda \right)}{<n>}\frac{c}{\tau \lambda^{3}}$ and $\frac{n^{2}\left(\lambda \right)}{{<n>}^{2}}\frac{1}{\lambda^{2}}$ for the Carr–Hermans approach, and $\frac{\tau \lambda^{5}}{\left(\frac{88}{15}\right)\pi^{3}n{\left(\lambda \right)}^{3}\frac{c}{N_{A}}{\left[\frac{dn}{dc}\left(\lambda \right)\right]}^{2}}$ and $\frac{\lambda^{2}}{n{\left(\lambda \right)}^{2}}$ for the Yeromonahos approaches).

Differences between the fit and true values for each diameter/length combination can be explained by the nonlinearities present in the full light scattering theory data, which are not accounted for by the linear fits of the different approaches. For example, the increased error in the calculations of both the diameter and mass–length ratios for large diameter fibers for all three approaches can be accounted for by the fact that the full light scattering theory plots are nonlinear for large diameters, especially at small wavelength values, as seen in Figure S8 in the Supporting Information. This nonlinearity results in a poor fit of the linear line, which results in inaccurate calculations of both the diameters and mass–length ratios for fibers with large diameters.

Furthermore, as seen in Figure S9 in the Supporting Information, the full light scattering theory plots are less linear for shorter fiber lengths for all three approaches, although the nonlinearity is not as obvious for the Yeromonahos plots. This nonlinearity results in the linear equations not being a good fit to the dataset, which then results in inaccuracies in the calculations of both the diameter and mass–length ratio for fibers with small lengths.

The only characteristic not explained by the nonlinearities of the full light scattering theory data are the areas of increased error for fibers of intermediate diameter using the corrected Yeromonahos approach. Both the Carr–Hermans and original Yeromonahos approach always underestimate the diameter, but by multiplying with the extra 2/3 factor in the denominator when solving for the diameter with the corrected Yeromonahos approach, the calculated value is increased so that it is closer to the actual diameter values, resulting in a smaller percentage error, as seen in Figure 5. While the corrected Yeromonahos approach still mostly underestimates the diameter and mass–length ratio, the areas of increased error in the centers of the plots in Figure 5 for the corrected Yeromonahos approach are caused by the calculated value for the diameter becoming larger than the actual values. Although this extra 2/3 factor decreases the percent error in the calculations of the diameter when compared to the original Yeromonahos approach, the factor was obtained erroneously, as described in Section S.2 of the Supporting Information. Therefore, there is no physical basis for using the “correction”.

The percent of the investigated diameter/length combinations in which each approach gives less than 10 percent error for each of the wavelength ranges investigated is shown in Table 3. As can be seen, the Carr–Hermans approach using a wavelength range of 500–800 nm provides less than 10 percent error in both the calculated diameter and the mass–length ratio for most of the diameter/length combinations investigated.

### 4.2. Experimental Analysis

Each of the investigated turbidimetric approaches were fit to experimental turbidimetry data for either a fibrin clot formed from 0.5 mg/mL fibrinogen and 0.1 NIH-U/mL thrombin, or a clot formed from 1 mg/mL fibrinogen, 0.1 NIH-U/mL thrombin, and 25 L-U/mL FXIIIa, in order to determine fiber diameter. Fibrin clots at identical concentrations were also prepared for STORM imaging, and fiber diameters were calculated from reconstructed STORM images and were used as a standard for evaluating the accuracy of the diameter values obtained using the turbidimetric approaches. STORM imaging and Simulated Emission Depletion Microscopy (STED), another super-resolution microscopy technique, have been previously used to determine fiber diameter from plasma [49,50,51] and from purified fibrinogen [52], providing fiber diameters over a range of 100–400 nm, matching those previously reported from SEM imaging [34]. However, super-resolution fluorescence imaging, unlike SEM imaging, does not require drying of the samples, which could cause changes in the morphology of the fibers.

As seen in Table 2, for the clot formed from 0.5 mg/mL fibrinogen, the diameter value obtained using STORM imaging is between the values obtained from experimental turbidimetry data using the Carr–Hermans approach and the corrected Yeromonahos approach over the range of 500–800 nm, accounting for the wavelength dependence of n and dn/dc. This supports the theoretical results that those two approaches, within that wavelength range, are the best at estimating the fiber diameter. Similarly, for the clot formed from 1 mg/mL fibrinogen, the Carr–Hermans approach using a wavelength range of 500–800 nm, accounting for the wavelength dependence of n and dn/dc, fell closest to the diameter value obtained using STORM imaging, supporting the simulated results that this approach is the most accurate.

As expected from the theoretical results, for both fibrin clot conditions analyzed, the values for fiber diameter are not as close to that obtained from imaging when using a wavelength range of 350–650 nm for any of the approaches, and the original Yeromonahos approach never estimates a fiber diameter that is similar to that obtained from imaging. Furthermore, both clot conditions show that the fiber diameter values calculated from turbidimetric analysis are further from those obtained from imaging when using constant values of n and dn/dc, rather than when accounting for their wavelength dependence, with the only exception being the Carr–Hermans approach using a wavelength range of 500–800 nm for the clot formed from 0.5 mg/mL fibrinogen. For this approach, the estimated diameter values using the Carr–Hermans approach and the corrected Yeromonahos approach with a wavelength range of 500–800 nm still fall within 20 nm of the value obtained from super-resolution fluorescence imaging when using a constant value of n and dn/dc, but this is not entirely unexpected, since n and dn/dc do not contain as large of a wavelength dependence within 500–800 nm, as shown in Figure S3 of the Supporting Information.

While the experimental evidence from these two clot conditions provides preliminary confirmation of the theoretical analysis, a full fibrinogen/thrombin concentration sweep is an important future step in order to determine if/where there is a point at which the theory no longer accurately represents the experimental data.

Although this work focused on the use of these turbidimetric approaches for the analysis of fibrin fibers, it can also be applied to other networks consisting of an assembly of cylindrical fibers, such as collagen, nanocellulose particles, and filamentous viruses, as long as they consist of dilute, long, thin, monodisperse cylindrical components of nanoscale diameter. If turbidimetry is used to analyze a filamentous network other than fibrin, Figures S6 and S7 in the Supporting Information can be used to determine which approaches may provide reasonable values for the expected range of diameters and lengths of the components within the network.

### 4.3. Comparing Theoretical and Experimental Results

Table 4 shows the percent error between the diameter value obtained from the experimental data using the turbidimetric approach and that obtained using STORM imaging (middle column) in comparison to the expected percent error according to the numerical simulations (right column) for the clot formed from 0.5 mg/mL fibrinogen. Table S3 in the Supporting Information shows these results for the clot formed from 1 mg/mL fibrinogen. The percent error values for the numerical simulations in Table 4 correspond to the percent errors shown in Figure 5 at a diameter value of 180 nm and length of 7 μm, because, for the 0.5 mg/mL clot, the experimentally determined mean diameter and length values were 181 nm (from STORM imaging) and 6.86 μm (from analysis of confocal images using IMARIS filament software), respectively. For the 1 mg/mL clot (Table S3), the numerical simulation percent errors used a diameter of 220 nm and a length of 6 μm, because the experimentally determined diameter and length were 218 nm and 6.27 μm, respectively. Remarkably, for both the 0.5 mg/mL and the 1 mg/mL samples, and for almost every approach, the numerical simulations predicted the percent error between the STORM diameter values and those obtained from turbidimetry to within a few percent.

The numerical simulations also predicted that all three approaches underestimate the diameter for fibers of these two thicknesses, which was true for all of the approaches except for the Carr–Hermans approach using a wavelength range of 500–800 nm, with the wavelength dependence of n and dn/dc being accounted for, for the clot formed from 0.5 mg/mL fibrinogen. The reason for the overestimation of the fiber diameter in this specific instance is unknown, but an 8% difference is still within the range of what is considered acceptable in Table 3. This suggests that the theory is able to reliably predict the accuracy of each of the turbidimetric approaches for fiber diameters of ~180 nm and ~220 nm, and that these predictions are likely also reliable at other diameters/lengths.

### 4.4. Theoretical Limitations

In analyzing the best fitting method, we compared values obtained from linear fits to the values used to calculate turbidity using the “full” light scattering theory. Because each of the linear fit equations are simplifications of the full light scattering theory, our work primarily assesses how reasonable these simplifications are. However, there are several possible limitations, even in the use of the full light scattering theory, that should be considered when comparing the theoretical turbidity data to that obtained from a real fibrin network.

The first limitation is that we are assuming that the fibers are Rayleigh–Gans–Debye scatterers. In order to be considered Rayleigh–Gans–Debye scatterers, the ratio of the index of refraction of the fibrinogen (n_p_) to the index of refraction of the surrounding medium (n_s_) should be close to one. Using the value of 1.6 for the index of refraction of most proteins [53], and the refractive index of HBS buffer (150 mM sodium chloride and 20 mM HEPES, pH 7.4) [42], n_p_/n_s_ is 1.196 ± 0.002 for the wavelength range being investigated (350–800 nm). Therefore, the criterion of being close to 1 is not fully met.

Another potential limitation is that turbidimetry assumes that the only attenuation mechanism is scattering by the fibers, and that no absorption takes place. At first glance, this may seem to be a poor assumption, since tryptophan, tyrosine, and phenylalanine have an absorption peak at approximately 280 nm, and fibrinogen contains a significant number of these amino acids. However, it has been shown that the absorption of these residues falls to zero (or close to it) prior to a wavelength of 350 nm [21,54], which is the lowest wavelength value used in our analysis. Thus, absorption plays little to no role in the loss of transmitted light, and so the assumption of most attenuation coming from scattering is reasonable. Furthermore, the absorption would likely be similar for polymerized and unpolymerized samples, so the change in transmitted light due to absorption would be removed when subtracting out the background.

Finally, the full light scattering theory for turbidimetry and all of the linear approaches use, in the calculations, the form factor for a thin rod. As the wavelength of light approaches the diameter, the justifications for using the thin rod form factor begin to weaken. As shown in Section S.7 of the Supporting Information, the error for all three turbidimetry methods increases above 200 nm. Interestingly, there was still good agreement between the Carr–Hermans (500–800 nm) turbidimetry results and the STORM-calculated diameters for the 1 mg/mL fibrinogen samples, with a diameter value of 218 nm. This is not completely surprising, as the numerical simulations predict a percent error of ~10% at this diameter. Nonetheless, this indicates that the approaches are still able to work reasonably well for diameters that are slightly above 200 nm. Further experimentation is needed to fully characterize how the thin rod approximation affects the ability to accurately determine fiber diameters that are much greater than 200 nm using turbidimetry, and so caution should be used if analyzing fibers with diameters in this range. 

### 4.5. Limitations in the Experimental Application of Turbidimetric Analysis

Not only are there limitations in the theory behind the turbidimetric approaches, but there are also potential limitations in the application of the theory to experimental data. One possible limitation in using turbidimetry for analyzing clots is that the light scattering theory equations (Equations (13) and (14)) assume that the fibers are dilute, and they therefore do not account for multiple scattering effects. The effects of multiple scattering were investigated by García et al. [33] who looked at the effect of changing optical pathlength on the estimated number of protofibrils per fiber. They found that the number of protofibrils is independent of the optical pathlength, which suggests that multiple scattering effects are insignificant. However, they only checked this for fibrinogen concentrations of 1 mg/mL and 3 mg/mL, and so this may not be the case if performing light scattering measurements on higher concentrations.

Furthermore, it is assumed by the equations that the fibers are homogeneous cylinders. However, the fibrin network can consist of fibers of varying lengths and diameters, as well as fibers that are not uniform in diameter along the length of the fiber. Since the light scattering equation uses a form factor that is averaged over all directions, the size parameters are also volume-averaged [26], and thus, this polydispersity is not accounted for. Therefore, it is important to keep in mind that the obtained diameter value from the turbidimetric approaches could be skewed by fibers with thick diameters, leading to an overestimation of the diameter. There can also be spatial heterogeneity present in the clot that is not accounted for, particularly if there are aggregates present. However, it has been found that there are stock solutions that do not contain aggregates and that create spatially uniform clots [33], so this problem can be largely avoided. Ferri et al. [32] also discussed the shortcomings of these assumptions, pointing out that although the single fibers might meet the criterion of being straight and randomly oriented, when the entire clot is considered, these assumptions may not be met, resulting in inaccuracies in the investigated turbidimetric approaches. In addition, recent work shows that the fibers themselves have non-uniform internal structures [55], which will cause additional deviations. While network heterogeneity did not appear to impact our experimental results compared to super-resolution fluorescence microscopy, it could play a role in the analysis of higher fibrinogen concentrations.

Despite these limitations, the turbidimetric approaches are still able to provide reasonable values for the diameter and mass–length ratio, as found through a comparison of the results with those from small-angle x-ray scattering [33], dynamic light scattering [26], and permeability measurements [25].

### 4.6. Practical Applications

Since the numerical simulations suggest that the Carr–Hermans approach using a wavelength range of 500–800 nm provides the most accurate results for the most combinations of fiber lengths and diameters, and that the experimentally obtained diameter value using that approach falls reasonably close to the value obtained through super-resolution imaging for the two investigated fibrinogen concentrations, we have provided an Excel analysis tool for fitting turbidimetry data with that approach. The fitting procedure accounts for the wavelength dependence of n and dn/dc, and it simply requires users to input turbidimetry data, along with the correct Cauchy relation coefficients for the particular buffer utilized. The program output values should be interpreted with caution, especially for any parameters falling outside the range where the Carr–Hermans approach has a theoretical accuracy within a 10% error (See Figure 5 and Figure 6, Figures S6 and S7).

## 5. Conclusions

In this work, we have evaluated the applicability of three commonly utilized turbidimetric approaches for determining the structures of individual elements within filamentous networks, with a specific focus on the analysis of fibrin fibers. The first important takeaway from the results is that it is necessary to account for the wavelength dependence of n and dn/dc, rather than using constant values. No matter which wavelength range is used, using a constant value for these factors considerably increases the percent error in the diameter and mass–length ratio calculations.

The results of the numerical simulations show that the utility of all three approaches is limited to certain values of fiber length, diameter, and wavelengths used for the turbidimetry measurements. As shown in Table 3, only the Carr–Hermans approach using a wavelength range of 500–800 nm, and the corrected Yeromonahos approach using a wavelength range of 500–800 nm, provide less than 10 percent error for over half of the diameter/length combinations investigated, with the Carr–Hermans approach providing less than 10 percent error for the most diameter/length combinations investigated (55.75% of combinations for diameter and 74.5% for the mass–length ratio). As seen in Figure S6 in the Supporting Information, the Carr–Hermans approach provides less than 10 percent error in the calculated diameter for fibers with diameters of 80–200 nm, and the corrected Yeromonahos approach provides less than 10 percent error in the calculated diameter for fibers with diameters of ~60–120 nm, as long as the length is greater than 1 µm. For fibers with diameters of 40–50 nm, the corrected Yeromonahos approach works best in the 350–650 nm wavelength range. Below 40 nm, all three approaches fail, as they give imaginary values for the diameter.

For the mass–length ratio, the Yeromonahos approaches provide less than 10 percent error for fibers with lengths in the range of 3–10 µm and diameters of ~10–140 nm, and the Carr–Hermans approach provides less than 10 percent error for fibers with lengths of 3–10 µm and diameters of ~10–200 nm. However, the Carr–Hermans approach always provides the least error in the mass–length ratio, with the Carr–Hermans approach using a wavelength range of 350–650 nm as being the best for fibers with diameters of less than ~140 nm, and the Carr–Hermans approach using a wavelength range of 500–800 nm being best for fibers with diameters of 140–200 nm. This can be seen in Figure S7 in the Supporting Information.

Although the corrected Yeromonahos approach does provide the most accurate results in calculating the diameter for some fiber diameter/length combinations, the Carr–Hermans approach is more often the most accurate, with less than 10 percent error for most diameter/length combinations investigated (69% of all the diameter/length combinations investigated). Even when the corrected Yeromonahos approach is better at calculating the diameter than the Carr–Hermans approach, it is only by a small difference in the percent error, with the largest difference being a 13.37% error, and the average difference being a 4.21% error. There is also no physical basis for the “correction” to the Yeromonahos approach, as is described in the Supporting Information Section S.2. Furthermore, the Carr–Hermans approach always provides the most accurate results in calculating the mass–length ratio. Therefore, under many situations, the Carr–Hermans equation, especially when using the 500–800 nm wavelength range, despite its flaws, provides the most accurate results. Since this approach was found to be best, a supplemental Excel analysis tool has been supplied for fitting “absorbance” versus wavelength data with the Carr–Hermans approach.

Preliminary experimental analysis provided further confirmation of the theoretical results. The diameter values obtained by fitting each of the investigated approaches to experimental turbidimetry data for a fibrin clot compared to the diameter value obtained using super-resolution fluorescence imaging supports the idea that the Carr–Hermans approach and the corrected Yeromonahos approach are able to reliably predict the fiber diameter when using a wavelength range of 500–800 nm, as the numerical simulations have suggested. It also suggests that using a wavelength range of 350–650 nm does not provide values that are as accurate, which matches the outcome of the numerical simulations. Furthermore, by comparing the predicted percent error in each of the approaches according to the numerical simulations to the percent error in the approaches when compared to the diameter values obtained through super-resolution imaging, it was shown that the theory was able to predict the percent error in the approaches within an accuracy of, on average, 3.9% for fibers with a diameter value of ~180 nm (Table 4), and 4% for fibers with a diameter of ~220 nm (Table S3).

To summarize, the commonly utilized approaches are, for the most part, able to provide accurate values of diameter, as long as the fibers have a length of 1–10 µm and a diameter of 50–200 nm, and the mass–length ratio can be determined with less than 10 percent error if the fibers have lengths of 3–10 µm and diameters of 10–200 nm. However, it is unclear whether turbidimetry analysis is able to provide accurate estimates at diameters that are far above 200 nm, as both full light scattering theory and the fitting approaches assume that the fibers are thin when compared to the wavelength values used for measurement.

Further work is needed in order to provide a complete analysis of full light scattering theory and the validity of turbidimetric approaches. The approach proposed by Ferri et al. [32] should be investigated further; full light scattering theory using a non-thin rod form factor could be investigated, and a more complete comparison of experimental data and theoretical approaches across multiple fiber lengths and diameters should be performed.

## Figures and Tables

**Table 1:** Average difference in percent error for diameter and mass–length ratio calculated from numerical simulations with constant values of n and dn/dc (values at 633 nm) and wavelength-dependence corrected values of n and dn/dc (for HBS buffer).

Average Difference in Percent Error of Values with Constant n and dn/dc and Wavelength Corrected n and dn/dc		
Approach	Diameter	Mass–Length Ratio
Carr–Hermans 350–650 nm	33%	5%
Carr–Hermans 500–800 nm	25%	5%
Original Yeromonahos 350–650 nm	24%	3%
Original Yeromonahos 500–800 nm	20%	4%
Corrected Yeromonahos 350–650 nm	28%	3%
Corrected Yeromonahos 500–800 nm	23%	4%

**Table 2:** Diameter values and uncertainty obtained by (A) fitting experimental data with the three turbidimetric approaches with the wavelength dependence of n and dn/dc accounted for, and (B) using constant values of n and dn/dc, and (C) using super-resolution fluorescence imaging for clots formed from 0.5 mg/mL fibrinogen and 0.1 NIH-U/mL thrombin in HBS buffer + 5 mM calcium chloride (middle column), and clots formed from 1 mg/mL fibrinogen, 0.1 NIH-U/mL thrombin, and 25 L-U/mL FXIIIa in HBS buffer + 5 mM calcium chloride (right column).

**(A) Turbidimetry-Based Diameters Using Wavelength-Corrected n and dn/dc**		
**Approach**	**Diameter (nm)**	
	**0.5 mg/mL** **Fibrinogen**	**1 mg/mL** **Fibrinogen**
Carr–Hermans 350–650 nm	153 ± 3	169 ± 2
Carr–Hermans 500–800 nm	196 ± 1	213 ± 2
Original Yeromonahos 350–650 nm	109 ± 2	113 ± 3
Original Yeromonahos 500–800 nm	142 ± 3	147 ± 3
Corrected Yeromonahos 350–650 nm	134 ± 3	139 ± 3
Corrected Yeromonahos 500–800 nm	174 ± 4	180 ± 3
**(B) Turbidimetry-Based Diameters Using Constant n and dn/dc**		
**Approach**	**Diameter (nm)**	
	**0.5 mg/mL** **Fibrinogen**	**1 mg/mL** **Fibrinogen**
Carr–Hermans 350–650 nm	126 ± 2	141 ± 2
Carr–Hermans 500–800 nm	176 ± 3	192 ± 2
Original Yeromonahos 350–650 nm	100 ± 2	105 ± 2
Original Yeromonahos 500–800 nm	135 ± 3	140 ± 3
Corrected Yeromonahos 350–650 nm	123 ±3	129 ± 3
Corrected Yeromonahos 500–800 nm	165 ± 4	172 ± 3
**(C) Diameters Using Super-Resolution Imaging**		
**Approach**	**Diameter (nm)**	
	**0.5 mg/mL** **Fibrinogen**	**1 mg/mL** **Fibrinogen**
Super-Resolution Imaging	181 ± 37	218 ± 52

**Table 3:** Percent of investigated diameter/length combinations in which each approach and wavelength range provided less than 10 percent error in the calculations of diameter and mass–length ratio.

% of Investigated Diameter/Length Combinations with Less than 10% Error		
Approach	Diameter	Mass-Length Ratio
Carr–Hermans 350–650 nm	49.75%	63%
Carr–Hermans 500–800 nm	55.75%	74.5%
Original Yeromonahos 350–650 nm	0%	46%
Original Yeromonahos 500–800 nm	0%	53.75%
Corrected Yeromonahos 350–650 nm	42.25%	46%
Corrected Yeromonahos 500–800 nm	51.25%	53.75%

**Table 4:** The percent error in the diameter values obtained experimentally using the turbidimetric fitting approaches (middle column), and the percent error predicted by the numerical simulations (right column), with (A) the wavelength dependence of n and dn/dc accounted for, and (B) using constant values of n and dn/dc. Clot formed from 0.5 mg/mL fibrinogen and 0.1 NIH-U/mL thrombin in HBS buffer + 5 mM calcium chloride.

**(A) Wavelength-Corrected n and dn/dc**		
	**Experimental** **Percent Error**	**Predicted** **Percent Error**
Carr–Hermans 350–650 nm	15%	15%
Carr–Hermans 500–800 nm	8%	4%
Original Yeromonahos 350–650 nm	40%	40%
Original Yeromonahos 500–800 nm	22%	28%
Corrected Yeromonahos 350–650 nm	26%	26%
Corrected Yeromonahos 500–800 nm	4%	12%
**(B) Constant n and dn/dc**		
	**Experimental** **Percent Error**	**Predicted** **Percent Error**
Carr–Hermans 350–650 nm	30%	30%
Carr–Hermans 500–800 nm	3%	15%
Original Yeromonahos 350–650 nm	45%	45%
Original Yeromonahos 500–800 nm	25%	33%
Corrected Yeromonahos 350–650 nm	32%	33%
Corrected Yeromonahos 500–800 nm	9%	17%

## Data Availability

Not applicable.

## Supplementary Materials

The following supporting information can be downloaded at: https://www.mdpi.com/article/10.3390/biom12060807/s1, Section S.1: Validity of approximations to full light scattering theory; Section S.2: Form factor correction in corrected Yeromonahos approach; Section S.3: Wavelength dependence of the refractive index and specific refractive index increment; Section S.4: Diameter values given by fits; Section S.5: Mass–length ratio values given by fits; Section S.6: Effects of fiber parameters on turbidity datasets; Section S.7: Larger diameter values; Section S.8: Experimental turbidimetry analysis; Section S.9: STORM imaging.
