# Supplementary material for: The Applicability of Current Turbidimetric Approaches for Analyzing Fibrin Fibers and Other Filamentous Networks
Source: Biomolecules. 2022 Jun 9;12(6):807. doi: 10.3390/biom12060807 (PMC9221518; doi:10.3390/biom12060807)
Supplement: Supplementary file 1 [file biomolecules-12-00807-s001.zip › Supplementary Files/Supporting Information.pdf]

Supporting Information for The Applicability of Current Turbidimetric Approaches for Analyzing Fibrin Fibers and Other Filamentous Networks

H. A. Belcher[1], K. Litwa[2], M. Guthold[3], N. E. Hudson[1*]

[1]Department of Physics, East Carolina University, Greenville, North Carolina 27858
[2]Department of Anatomy & Cell Biology, East Carolina University, Greenville, North Carolina 27858
[3]Department of Physics, Wake Forest University, Winston-Salem, North Carolina 27109

*Corresponding Author: hudsonn16@ecu.edu

S.1: Validity of Approximations to Full Light Scattering Theory

The three fitting approaches make several approximations in order to simplify full light scattering theory (Equations 13-14). The first approximation made by all three approaches is that the fiber length is infinite (L>>λ). This allows for the simplification of the term $Si(qL) \approx \frac{\pi}{2}$, since if L goes to infinity, $Si(qL) = \int_0^\infty \frac{sinx}{x} dx = \frac{\pi}{2}$. It also allows for the simplification that $\left[\frac{\sin\left(\frac{qL}{2}\right)}{\frac{qL}{2}}\right]^2 \approx 0$ since the denominator approaches infinity. However, in some cases, L~λ, making L>>λ a poor assumption. As Ryan et. al. [1] show, the average length can be as low as 0.3 μm, which is comparable to the lowest wavelength values used (0.35 μm) in some fitting approaches.

The next assumption made by all three approaches is that $\frac{qd}{2} \ll 1$, which would be true if the fibers are very thin (d<<λ). This allows for the Bessel function to be expanded and keeping the first two terms, $S_{sec} = 1 - \frac{(qd)^2}{16} + \frac{(qd)^4}{1024}$. Then the $\frac{(qd)^4}{1024}$ term can be neglected since if $\frac{qd}{2} \ll 1$, $\frac{(qd)^4}{1024}$ would also be much less than 1. To assess whether $\frac{qd}{2} \ll 1$, we inserted realistic values into the term. For the lowest diameter value we investigated, 10 nm, $\frac{q_{max}d}{2} = 0.18$ where $q_{max} = \frac{4\pi}{350nm}\sin\left(\frac{\pi}{2}\right)$. In this case, $\frac{q_{max}d}{2}$ is much less than 1. For the largest diameter value investigated, 200 nm, $\frac{q_{max}d}{2} = 3.59$, which is greater than 1, making the assumption invalid. This may explain the increase in error for larger diameter values, especially when using lower wavelength ranges. However, keeping the $\frac{(qd)^4}{1024}$ term in this Taylor expansion would lead to a complicated polynomial equation that cannot be used to accurately determine the parameters.

While the above two assumptions are not always true, these simplifications of the full light scattering theory Rayleigh ratio are necessary in order to get linear equations for fitting experimental turbidity datasets.

Carr and Hermans also use the assumption that $\frac{23}{77}\pi^2 n^2 d^2 \frac{1}{\lambda^2} \ll 1$ in order to make the approximation $\left[1 - \frac{23}{77}\pi^2 n^2 d^2 \frac{1}{\lambda^2}\right]^{-1} = 1 + \frac{23}{77}\pi^2 n^2 d^2 \frac{1}{\lambda^2}$ by keeping the first two terms of the Taylor expansion. The third term in the Taylor expansion would be $\frac{529}{5929}\pi^4 n^4 \frac{d^4}{\lambda^4}$, which for a diameter of 100 nm and a wavelength of 350 nm would be 0.19. Compared to the previous term in the Taylor expansion, $\frac{23}{77}\pi^2 n^2 d^2 \frac{1}{\lambda^2}$, which would be 0.44 for the same diameter and wavelength, this term is not negligible, as assumed. However, Ferri [2] argues that this approximation balances with the previous two approximations, resulting in the linear behavior

continuing beyond its limits of applicability. Our fitting results confirm this, since the Carr-Hermans approach contains less error than the original Yeromonahos approach for all of the diameter/length combinations explored, when the original Yeromonahos approach makes the previous two approximations, but not this one.

We attempted to improve the current approaches by applying fewer simplifications to full light scattering theory, with the hope that it would provide more accurate estimates of the diameter and mass-length ratio. Among the attempted improvements, we tried keeping the sine term in P_{rod}, while making the above simplification that $\frac{2Si(qL)}{qL} \approx \frac{\pi}{qL}$, so that $P_{rod} = \frac{\pi}{qL} - \left[\frac{\sin\left(\frac{qL}{2}\right)}{\frac{qL}{2}}\right]^2$. We also attempted to keep $S_{sec} = 1 - \frac{(qd)^2}{16} + \frac{(qd)^4}{1024}$, instead of neglecting the last term, as described above. Additionally, we tried keeping one more term in the Taylor expansion used by Carr and Hermans so that $\left[1 - \frac{23}{77}\pi^2 n^2 d^2 \frac{1}{\lambda^2}\right]^{-1} = 1 + \frac{23}{77}\pi^2 n^2 d^2 \frac{1}{\lambda^2} + \frac{529}{5929}\pi^4 n^4 d^4 \frac{1}{\lambda^4}$. Finally, we tried adjusting the Carr-Hermans equation by the 2/3 term Yeromonahos added in their correction. Unfortunately, none of these attempts were successful at creating a more accurate fitting approach, as they either led to polynomial equations that were too complicated to fit to the data, imaginary values of diameter, or just increased error in the calculations.

S.2: Form Factor Correction in Corrected Yeromonahos Approach

It is argued by Yeromonahos [3] that a correction is needed to their original equation (Equation 18 in the main text of this paper) in order to account for the form factor being averaged over the entire solid angle, leading to the corrected Yeromonahos equation (Equation 19 in the main text of this paper). However, there are several apparent errors in the derivation of obtaining the corrected equation (equations 10.17-10.26 in reference 3). These errors will be briefly discussed. To reduce confusion, all equations from reference 3 will begin with "10", all equations from the main text of this paper will be referred to by a whole number, and all equations that we are correcting in the supplement will begin with "S".

The first error is that according to reference 3, integrating Equation 10.16 leads to the equation:

$$P(\theta) = \frac{1}{4k_0L^2}\left[-2Csc^2\left(\frac{\theta}{2}\right) + 2\cos\left(2k_0Lsin\left(\frac{\theta}{2}\right)Csc^2\left(\frac{\theta}{2}\right)\right] + \frac{k_0r^2}{4k_0L^2}\left[4 - \cos\left(2k_0Lsin\left(\frac{\theta}{2}\right)Csc^2\left(\frac{\theta}{2}\right) + cos\theta\cos\left(2k_0Lsin\frac{\theta}{2}\right)Csc^2\left(\frac{\theta}{2}\right)\right] - \frac{k_0r^2}{4k_0L^3}Csc\left(\frac{\theta}{2}\right)\sin\left(2k_0Lsin\frac{\theta}{2} + \frac{1}{k_0L}\left[Csc\left(\frac{\theta}{2}\right)SinIntegral\left(2k_0Lsin\frac{\theta}{2}\right)\right] + \frac{k_0r^2}{4k_0L}\left[-2Csc\left(\frac{\theta}{2}\right)SinIntegral\left(2k_0Lsin\frac{\theta}{2}\right) + cos\theta Csc\left(\frac{\theta}{2}\right)SinIntegral\left(2k_0Lsin\frac{\theta}{2}\right)\right]. \quad [S.1]$$

However, integrating Equation 10.16 using Mathematica 12.1 (Wolfram, Champaign, IL) actually provides the equation:

$$P(\theta) = \frac{1}{4k_0^2L^3}\left[4L\left(k_0^2r^2 - Csc^2\left(\frac{\theta}{2}\right)\right)\sin^2\left(k_0Lsin\left(\frac{\theta}{2}\right)\right) + k_0r^2\left(2k_0L - Csc\left(\frac{\theta}{2}\right)\sin\left(2k_0Lsin\left(\frac{\theta}{2}\right)\right)\right) + 2k_0L^2(2 - k_0^2r^2 + k_0^2r^2cos\theta)Csc\left(\frac{\theta}{2}\right)SinIntegral\left(2k_0Lsin\left(\frac{\theta}{2}\right)\right)\right]. \quad [S.2]$$

which does not seem to simplify to Equation S.1

Next, according to Equation 10.22 in reference 3, $\tau = A\int_0^{\pi} P(\theta)sin\theta(\cos^2\theta + 1)d\theta$ where A is defined as: $A = \frac{2\pi^2 n_s^2}{\lambda^4} m_f^2 \left(\frac{dn}{dc}\right)^2$. However, based on Equation 10.21, $A = \frac{4\pi^3 n_s^2 c}{\lambda^4} m_f \left(\frac{dn}{dc}\right)^2$, and based on another source [2], A should also include Avogadro's number. Therefore, the correct form of A is given by:

$$A = \frac{4\pi^3 n_s^2 c}{N_A\lambda^4} m_f \left(\frac{dn}{dc}\right)^2. \quad [S.3]$$

An analytical solution to Equation 10.22 is then given; however, using the correct value of P(θ) as given in Equation S.2 above, the integral cannot be solved analytically using Mathematica 12.1 (Wolfram, Champaign, IL).

Then, based on Equation 10.24 in reference 3, Equation 10.25 should be

$$\int_0^{\pi} P(\theta)\sin\,\theta(\cos^2\theta+1)d\theta = 4\pi\left(\frac{77-23k_0^2r^2}{105k_0L}\right) \qquad [S.4]$$

where k_0 is squared, unlike as given in Equation 10.25 in reference 3.

Finally, combining Equations 10.22 and Equations 10.25 do not provide Equation 10.26 as suggested. Using the equations as given in reference 3, the solution would instead be:

$$\tau\lambda^5 = \pi^2 n\mu^2 L\left(\frac{dn}{dc}\right)^2\frac{44}{15}\left[\lambda^2-\frac{46}{77}r^2\pi n\lambda\right]. \qquad [S.5]$$

However, Equation S.5 uses the incorrect value of A. Using the correct value of A in Equation S.3 along with erroneous Equation 10.25 provides:

$$\tau\lambda^5 = \frac{\pi^3 cn\mu}{N_A}\left(\frac{dn}{dc}\right)^2\frac{88}{15}\left[\lambda^2-\frac{46}{77}r^2\pi n\lambda\right]. \qquad [S.6]$$

and using the correct value of A as well as the k_0 value being squared as in Equation S.4, the solution would then be:

$$\tau\lambda^5 = \frac{\pi^3 cn\mu}{N_A}\left(\frac{dn}{dc}\right)^2\frac{88}{15}\left[\lambda^2-\frac{92}{77}\pi^2r^2n^2\right] \qquad [S.7]$$

which is actually the original Yeromonahos equation (Equation 18 in the main text of this paper). However, none of these results provide the "corrected" Yeromonahos equation (Equation 19 in the main text of this paper), as reference 3 suggests. Thus, the factor of 2/3 that differentiates main text Equation 18 from Equation 19 has no physical basis.

Curiously, although Equation 19 has no physical basis, it does provide improved results compared to the original Yeromonahos equation (Equation 18 in the main text of this paper). This is due to the fact that the original Yeromonahos approach always underestimates the diameter and by adding this extra 2/3 factor into the equation, the diameter becomes $d = 2\sqrt{\frac{-(yintercept)}{slope*(\frac{184}{231}\pi^2)}}$ instead of $d = 2\sqrt{\frac{-(yintercept)}{slope*(\frac{184}{154}\pi^2)}}$, decreasing the value in the denominator and thus increasing the calculated diameter value, making it closer to the actual values.

S.3: Wavelength Dependence of the Refractive Index and Specific Refractive Index Increment

The effect of using a constant value for n and dn/dc in the fitting approaches was compared with using wavelength dependence-corrected values for n and dn/dc, and the results are shown in Figure S.1 for the diameter and S.2 for the mass-length ratio. The original dataset created using full light scattering theory was always corrected for the wavelength dependence of n and dn/dc regardless of whether the fitting approach was. The wavelength correction equations given in the methods section of the main text (Equations 20 and 21) for HBS buffer were used to calculate values of n and dn/dc at each wavelength. In the situations where we used a single n and dn/dc value for the fitting, we chose the values corresponding to 633 nm (n=1.3344, dn/dc=0.1901), because that is the wavelength originally utilized by Carr and Hermans in their seminal paper [4]. The error was also determined using the spectrally averaged values of n and dn/dc for both wavelength ranges, although that yielded very similar results as using the 633 nm values and is therefore not shown.

As can be seen in Figures S.1 and S.2, using a constant value for n and dn/dc results in significantly increased error in the calculations of the diameter and mass-length ratio compared to when accounting for the wavelength dependence of the two terms. This is the case for all three approaches, at both wavelength ranges investigated.

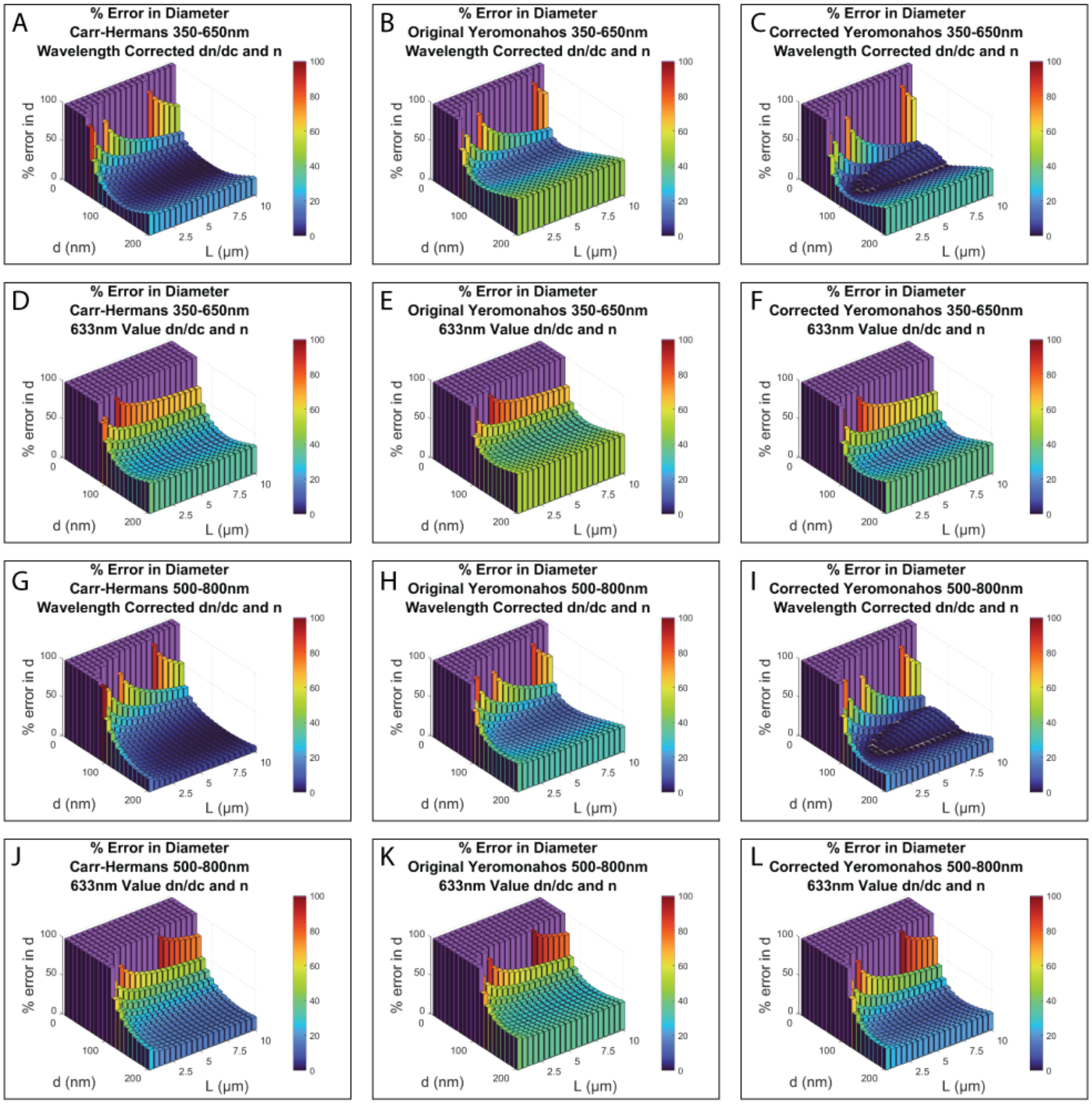

Fig. S.1: The percent error in the diameter between the value used to create the full light scattering theory dataset (using Equations 13-14) and the values obtained using the Carr-Hermans (left column), original Yeromonahos (middle column), and corrected Yeromonahos (right column) approaches (Equations 17-19, respectively), for wavelength ranges of 350-650 nm and 500-800 nm, using wavelength corrected values of n and dn/dc (A-C, G-I), and the constant values of n and dn/dc at 633 nm (D-F, J-L). (d=10-200 nm, L=0.5-10 μm, c=0.0001 g/cm^3, μ=4.73*10^{12} Da/cm, values of dn/dc and n for HBS buffer: 150 mM sodium chloride, 20 mM HEPES, pH 7.4; purple bars represent imaginary values and were assigned 100% error for plotting purposes)

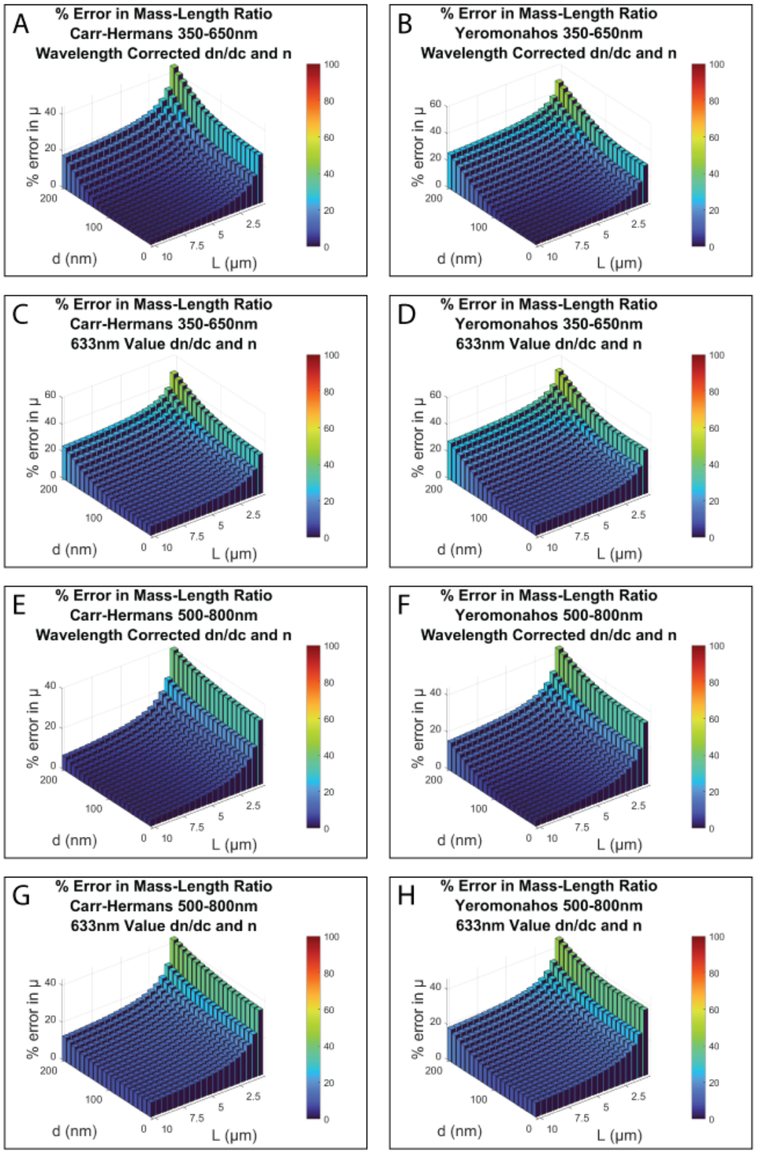

Fig. S.2: The percent error in the mass-length ratio between the value used to create the full light scattering theory dataset (using Equations 13-14) and the value obtained with the Carr-Hermans (left column), and original and corrected Yeromonahos (right column) approaches (Equations 17-19, respectively), for wavelength ranges of 350-650 nm and 500-800 nm, using wavelength corrected values of n and dn/dc (A-B, E-F), and the constant values of n and dn/dc at 633 nm (C-D, G-H). (d=10-200 nm, L=0.5-10 μm, c=0.0001 g/cm^3, μ=4.73*10^{12} Da/cm, values of dn/dc and n for HBS buffer: 150 mM sodium chloride, 20 mM HEPES, pH 7.4)

It is argued by García, et. al. [5] that it is not as necessary to correct for the wavelength dependence of n and dn/dc if using a wavelength range of 500-800 nm, because as can be seen in Figure S.3, there is a lesser wavelength dependence of the parameters in that wavelength range than there is at lower wavelengths. As shown in Table 1 of the main text, there is in fact less added error in the calculations of the diameter when using constant values in the 500-800 nm wavelength range than there is when using the 350-650 nm wavelength range. However, there is still a considerable amount of added error when using constant values of n and dn/dc in the 500-800 nm wavelength range, and there is actually more added error in the

mass-length ratio in that wavelength range for the Yeromonahos approaches. Therefore, it is best to always correct for the wavelength dependence of n and dn/dc in order to limit the excess error in the calculations.

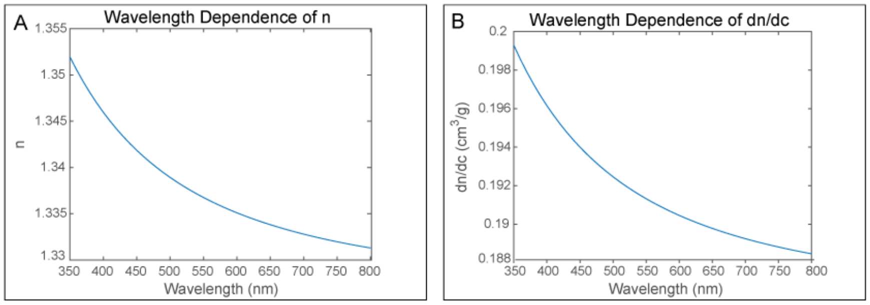

Fig. S.3: The wavelength dependence of A) n and B) dn/dc for fibrin fibers in HBS buffer (150 mM sodium chloride, 20 mM HEPES, pH 7.4) from 350-800 nm.

When accounting for the wavelength dependence of n and dn/dc, the plots need to be altered so that the equations using the slope and y-intercept to solve for the diameter and mass-length ratio do not include the terms n(λ) and dn/dc(λ), as they are now dependent upon the wavelength and therefore cannot be applied to the entire plot.

Following the work of Ferri et. al. [2], the Carr-Hermans equation rewritten to account for the wavelength dependence of n and dn/dc becomes:

$$\frac{[(dn/dc(\lambda))]^2}{\langle dn/dc\rangle^2}\frac{n(\lambda)}{\langle n\rangle}\frac{c}{\tau\lambda^3} = N_A\left[\frac{88}{15}\pi^3\langle n\rangle\langle\frac{dn}{dc}\rangle^2\right]^{-1}\frac{1}{\mu}\left[1+\frac{23}{77}\pi^2\langle n\rangle^2 d^2\frac{n^2(\lambda)}{\langle n\rangle^2}\frac{1}{\lambda^2}.\right. \quad \text{[S.8]}$$

Then instead of using $\frac{c}{\tau\lambda^3}$ vs. $\frac{1}{\lambda^2}$ for the Carr-Hermans approach, the plots should now be plotted as y' vs. x', where [2]:

$$y' = \frac{\left[\left(\frac{dn}{dc}\right)(\lambda)\right]^2}{<\frac{dn}{dc}>^2}\frac{n(\lambda)}{<n>}\frac{c}{\tau\lambda^3} \quad \text{[S.9]}$$

$$x' = \frac{n^2(\lambda)}{<n>^2}\frac{1}{\lambda^2} \quad \text{[S.10]}$$

where <n> and <dn/dc> are the spectral averages of the refractive index and specific refractive index, respectively, given by:

$$< n > = \frac{1}{\lambda_2-\lambda_1}\int_{\lambda_1}^{\lambda_2} n(\lambda)d\lambda \quad \text{[S.11]}$$

$$< dn/dc > = \frac{1}{\lambda_2-\lambda_1}\int_{\lambda_1}^{\lambda_2}\frac{dn}{dc}(\lambda)d\lambda \quad \text{[S.12]}$$

As seen in Figure S.4, this also results in plots that are more linear compared to those of y vs. x as described in the main text, especially for larger diameters. Now, the mass-length ratio and diameter can be solved for by:

$$\mu = \frac{N_A}{\left(\frac{88}{15}\right)\pi^3<n><\frac{dn}{dc}>^2(yintercept)} \quad \text{[S.13]}$$

$$d = \sqrt{\frac{6776\pi<\frac{dn}{dc}>^2\mu(slope)}{345N_A<n>}} \quad \text{[S.14]}$$

Then for both the original and corrected Yeromonahos approaches, instead of plotting $\tau\lambda^5$ vs. λ^2, the plots should instead be plotted as y' vs. x', where [5]:

$$y' = \frac{\tau\lambda^5}{A'} \quad \text{[S.15]}$$

$$x' = \frac{\lambda^2}{n(\lambda)^2} \quad \text{[S.16]}$$

where $A' = \left(\frac{88}{15}\right)\pi^3 n(\lambda)^3 \frac{c}{N_A}\left[\left(\frac{dn}{dc}\right)(\lambda)\right]^2$. As can be seen in Figure S.4, this change does not affect the linearity of the plots, but it does remove the n(λ) and dn/dc(λ) from the equations used to solve for the diameter and mass-length ratio, which are now:

$$\mu = slope \qquad [S.17]$$

$$d = 2\sqrt{\frac{-(yintercept)}{(slope)B'}} \qquad [S.18]$$

where for the original Yeromonahos approach, $B' = \frac{184}{154}\pi^2$, and for the “corrected” Yeromonahos approach, $B' = \frac{184}{231}\pi^2$.

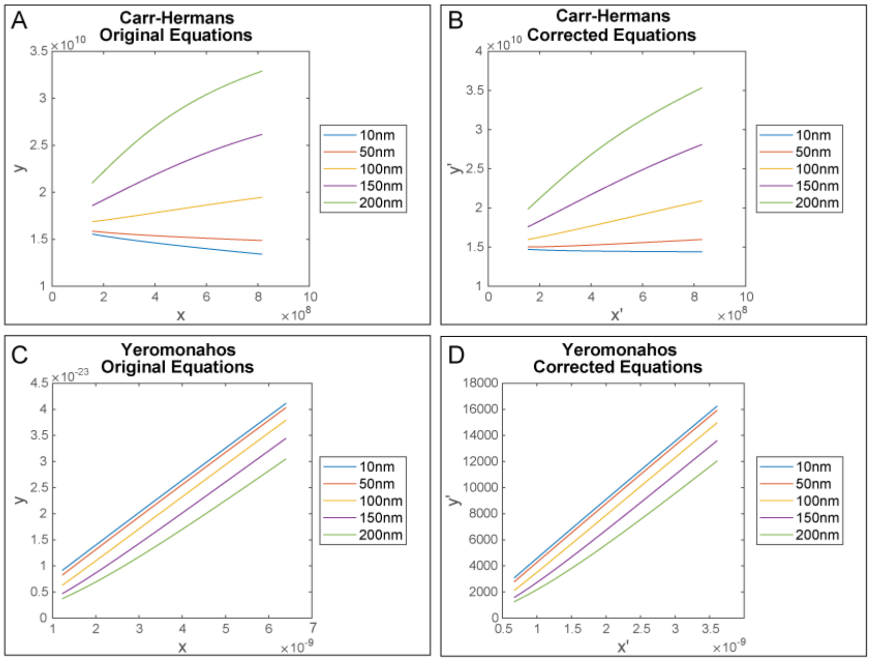

Fig. S.4: A theoretical turbidity dataset created using full light scattering theory (Equations 13-14) plotted as A) $\frac{c}{\tau\lambda^3}$ as y and $\frac{1}{\lambda^2}$ as x, B) $\frac{\left[\left(\frac{dn}{dc}\right)(\lambda)\right]^2}{\langle\frac{dn}{dc}\rangle^2}\frac{n(\lambda)}{\langle n\rangle}\frac{c}{\tau\lambda^3}$ as y’ and $\frac{n^2(\lambda)}{\langle n\rangle^2}\frac{1}{\lambda^2}$ as x’, C) $\tau\lambda^5$ as y and λ^2 as x, D) $\frac{\tau\lambda^5}{A'}$ as y’ and $\frac{\lambda^2}{n(\lambda)^2}$ as x’, for diameters of 10, 50, 100, 150, and 200 nm. (L=5 μm, c=0.0001 g/cm^3, μ=4.73*10^{12}Da/cm, λ=350-800 nm, values of n and dn/dc for HBS buffer: 150 mM sodium chloride, 20 mM HEPES, pH 7.4)

The methods for evaluating the different approaches while accounting for the wavelength dependence of n and dn/dc can be seen in Figure S.5.

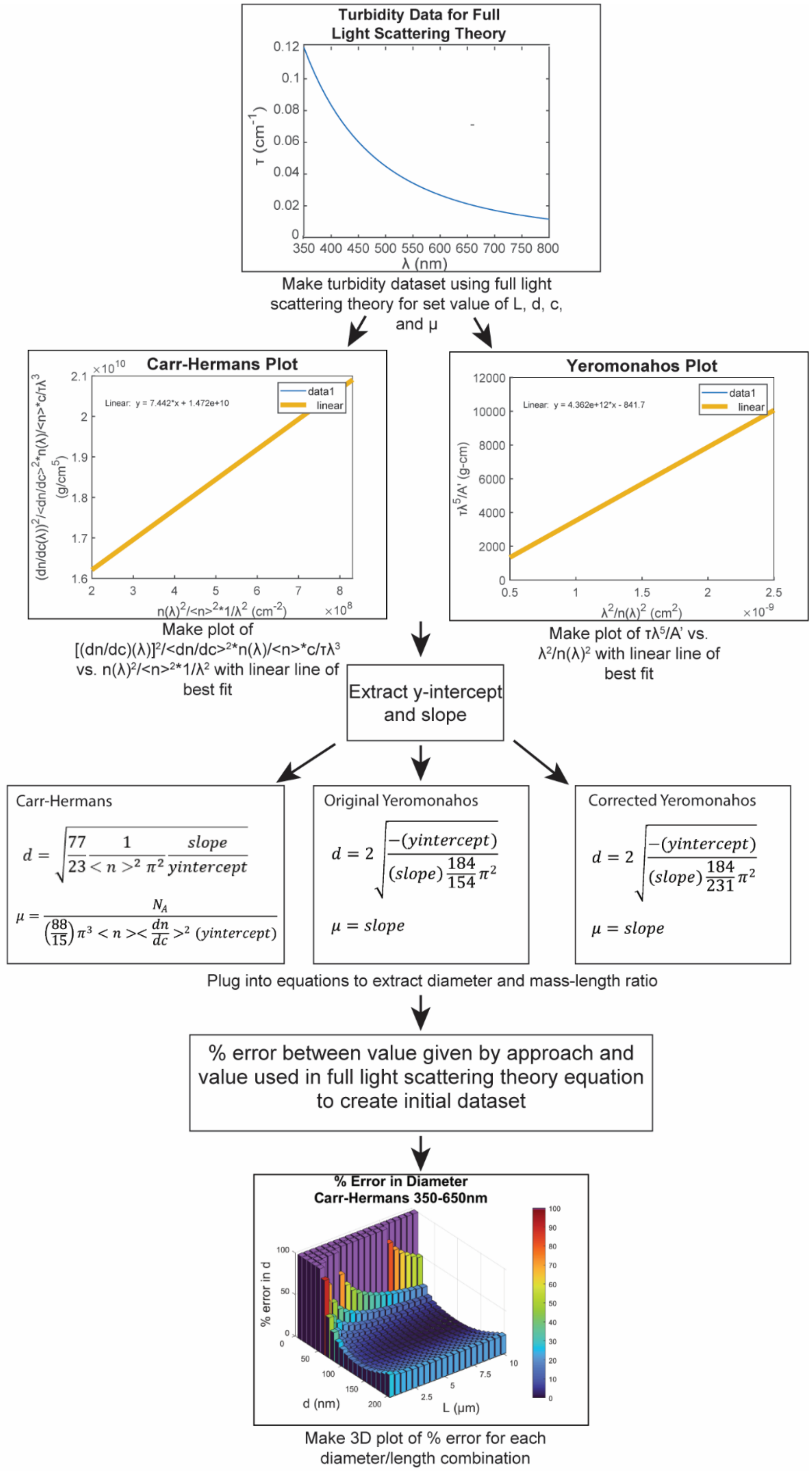

Fig. S.5: An outline of the methods for determining the percent error in each approach, corrected for the wavelength dependence of n and dn/dc.

S.4: Diameter Values Given by Fits

Table S.1 gives the actual values of diameter calculated by the approaches for lengths of 0.5 and 5 μm and diameters of 50 and 150 nm. For all three of the approaches at both wavelength ranges, when the length is small (0.5 μm) and the diameter is small (50 nm), fitting the full light scattering theory data with Equations 17-19 provides imaginary values for the diameter, which we assigned a value of 100% error for plotting purposes. This is shown by the purple bars in Figure 5 for small diameter values.

The amount of error in the estimates of diameter by the approaches then decreases for increasing diameter, as well as increasing length, other than an increase in the error by the original and corrected Yeromonahos approaches for diameters of 150 nm with lengths of 5 μm, as compared to the error for a diameter of 50 nm and length of 5 μm.

A

**Carr-Hermans**	Input L (μm)	Input d (nm)	d from Fit (nm)	% Error in d
350-650 nm	0.5	50	Imaginary	*100
	0.5	150	127.52	14.99
	5	50	44.37	11.26
	5	150	139.04	7.31
500-800 nm	0.5	50	Imaginary	*100
	0.5	150	118.15	21.23
	5	50	35.35	29.30
	5	150	145.84	2.77

B

**Original Yeromonahos**	Input L (μm)	Input d (nm)	d from Fit (nm)	% Error in d
350-650 nm	0.5	50	Imaginary	*100
	0.5	150	96.20	35.87
	5	50	41.39	17.22
	5	150	101.96	32.03
500-800 nm	0.5	50	Imaginary	*100
	0.5	150	98.62	34.25
	5	50	33.10	33.80
	5	150	116.64	22.24

C

**Corrected Yeromonahos**	Input L (μm)	Input d (nm)	d from Fit (nm)	% Error in d
350-650 nm	0.5	50	Imaginary	*100
	0.5	150	117.83	21.45
	5	50	50.69	1.38
	5	150	124.88	16.75
500-800 nm	0.5	50	Imaginary	*100
	0.5	150	120.78	19.48
	5	50	40.53	18.94
	5	150	142.86	4.76

Table S.1: Values of diameter (input d) at lengths of 0.5 μm and 5 μm (input L) used in the full light scattering theory equation (Equations 13-14) to create the turbidity dataset, compared to the value given out from a linear line of best fit to that data (d from fit) using the A) Carr-Hermans approach (Equation 17), B) Original Yeromonahos approach (Equation 18), and C) Corrected Yeromonahos approach (Equation 19), and the calculated percent error between the input value and the value given by the approach (% error in d). ($c=0.0001$ g/cm^3; $\mu=4.73*10^{12}$ Da/cm; imaginary diameters assigned a value of 100% error)

Figure S.6 shows which approach is best for determining the diameter for each fiber length/diameter combination (shown by the color), as well as the approaches that result in less

than ten percent error for that length/diameter combination (shown by the letters). If no approaches result in less than ten percent error for a length/diameter combination, no information is given.

% Error in Diameter											
		Length (µm)									
		1	2	3	4	5	6	7	8	9	10
Diameter (nm)	20										
	40					k	k	k	k	k	ck
	60		k	k	ck	ckK	ckK	ckK	ckK	ckK	cCkK
	80		ck	ckK	ckK	cCkK	cCkK	cCkK	cCkK	cCkK	cCkK
	100	k	ckK	cCkK	cCkK	cCkK	cCkK	cCkK	cCkK	cCkK	cCkK
	120	c	cCkK	cCkK	cCkK	cCkK	cCkK	cCkK	cCkK	cCkK	cCkK
	140	c	cCK	cCk	cCK	cCK	cCK	cCK	cCK	cCK	cCk
	160		CK	CK	cCK	cCK	cCK	cCK	cCK	cCK	cCK
	180		C	C	C	C	C	C	C	C	C
	200		C	C	C	C	C	C	C	C	C

Best Approach Legend	
(light blue)	Carr-Hermans 350-650nm
(blue)	Carr-Hermans 500-800nm
(light green)	Original Yeromonahos 350-650nm
(dark green)	Original Yeromonahos 500-800nm
(light orange)	Corrected Yeromonahos 350-650nm
(orange)	Corrected Yeromonahos 500-800nm

Approaches Less than 10% Legend	
c	Carr-Hermans 350-650nm
**C**	Carr-Hermans 500-800nm
y	Original Yeromonahos 350-650nm
**Y**	Original Yeromonahos 500-800nm
k	Corrected Yeromonahos 350-650nm
**K**	Corrected Yeromonahos 500-800nm

Fig. S.6: Summary of best approaches/approaches that provide less than 10% error for determining fibrin diameter at lengths of 1-10 µm and diameters of 20-200 nm. The color shows the best approach for that diameter/length combination and the letters show the approaches that provide less than 10% error for that diameter/length combination.

S.5: Mass-Length Ratio Values Given by Fits

Table S.2 shows the actual mass-length ratio values calculated by the approaches for lengths of 0.5 and 5 µm and diameters of 50 and 150 nm. As can be seen, for all three approaches the percent error in the mass-length ratio increases with increasing diameter and decreases with increasing length.

A Carr-Hermans	Input L (μm)	Input d (nm)	Input μ (Da/cm * 10^{-12})	μ from Fit (Da/cm * 10^{-12})	% Error in μ
350-650 nm	0.5	50	4.73	3.45	27.06
	0.5	150	4.73	3.14	33.62
	5	50	4.73	4.52	4.44
	5	150	4.73	4.31	8.88
500-800 nm	0.5	50	4.73	3.20	32.35
	0.5	150	4.73	3.05	35.52
	5	50	4.73	4.46	5.71
	5	150	4.73	4.43	6.34

B Yeromonahos	Input L (μm)	Input d (nm)	Input μ (Da/cm * 10^{-12})	μ from Fit (Da/cm * 10^{-12})	% Error in μ
350-650 nm	0.5	50	4.73	3.40	28.12
	0.5	150	4.73	2.93	38.05
	5	50	4.73	4.50	4.86
	5	150	4.73	3.97	16.07
500-800 nm	0.5	50	4.73	3.14	33.62
	0.5	150	4.73	2.96	37.42
	5	50	4.73	4.46	5.71
	5	150	4.73	4.23	10.57

Table S.2: Values of mass-length ratio (input μ) at diameters of 50 and 150 nm (input d) and lengths of 0.5 μm and 5 μm (input L) used in the full light scattering theory equation (Equations 13-14) to create the turbidity dataset, compared to the value given out from a linear line of best fit to that data (μ from fit) using the A) Carr-Hermans approach (Equation 17), and B) original and corrected Yeromonahos approaches (Equations 18-19), and the calculated percent error between the input value and value given by the fit (% error in μ). (c=0.0001 g/cm^3)

Figure S.7 shows which approach is best for determining the mass-length ratio for each fiber length/diameter combination (shown by the color), as well as the approaches that result in less than ten percent error for that length/diameter combination (shown by the letters). If no approaches result in less than ten percent error for a length/diameter combination, no information is given.

% Error in Mass-Length Ratio

Diameter (nm)	Length (μm): 1	2	3	4	5	6	7	8	9	10
20		cy	cCyY	cCyY	cCyY	cCyY	cCyY	cCyY	cCyY	cCyY
40		cy	cCyY	cCyY	cCyY	cCyY	cCyY	cCyY	cCyY	cCyY
60		c	cCyY	cCyY	cCyY	cCyY	cCyY	cCyY	cCyY	cCyY
80		c	cCyY	cCyY	cCyY	cCyY	cCyY	cCyY	cCyY	cCyY
100			cCY	cCyY	cCyY	cCyY	cCyY	cCyY	cCyY	cCyY
120			cC	cCY	cCY	cCyY	cCyY	cCyY	cCyY	cCyY
140			C	cC	cCY	cCY	cCY	cCY	cCY	cCY
160			C	C	C	cC	cC	cC	cCY	cCY
180				C	C	C	C	C	C	C
200					C	C	C	C	C	C

Best Approach Legend	
(light blue)	Carr-Hermans 350-650nm
(blue)	Carr-Hermans 500-800nm
(light orange)	Yeromonahos 350-650nm
(orange)	Yeromonahos 500-800nm

Approaches Less than 10% Legend	
c	Carr-Hermans 350-650nm
**C**	Carr-Hermans 500-800nm
y	Yeromonahos 350-650nm
**Y**	Yeromonahos 500-800nm

Fig. S.7: Summary of best approaches/approaches that provide less than 10% error for determining fibrin mass-length ratio at lengths of 1-10 μm and diameters of 20-200 nm. The color shows the best approach for that diameter/length combination and the letters show the approaches that provide less than 10% error for that diameter/length combination.

S.6: Effects of Fiber Parameters on Turbidity Datasets

Effect of Fiber Diameter:

The effect of changing diameter on full light scattering theory (Equations 13-14) can be seen in Figure S.8. As can be seen, the full light scattering theory plots are less linear for fibers with increasing diameters. The implications of this nonlinearity on the percent error in the three fitting approaches (Equations 17-19) are described in the discussion section of the main text.

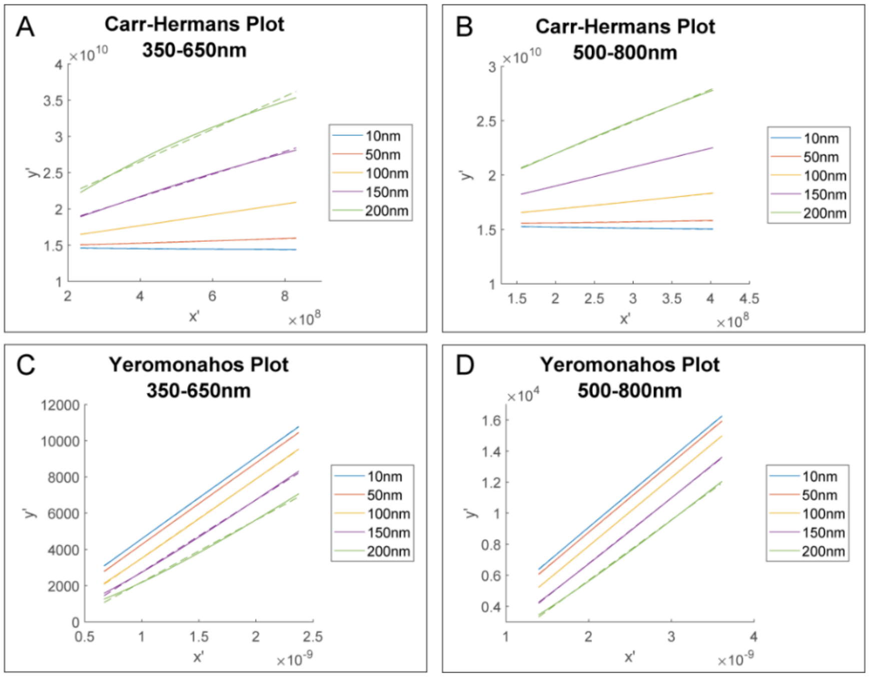

Fig. S.8: A theoretical turbidity dataset created using full light scattering theory (Equations 13-14) for diameters of 10, 50, 100, 150, and 200 nm plotted in A) the Carr-Hermans method of $\frac{\left(\frac{dn}{dc}(\lambda)\right)^2}{<\frac{dn}{dc}>^2}\frac{n(\lambda)}{<n>}\frac{c}{\tau\lambda^3}$ vs. $\frac{n^2(\lambda)}{<n>^2}\frac{1}{\lambda^2}$ for the 350-650 nm wavelength range and B) 500-800 nm wavelength range and C) the Yeromonahos method of $\frac{\tau\lambda^5}{A'}$ vs. $\frac{\lambda^2}{n(\lambda)^2}$ for the 350-650 nm wavelength range and D) 500-800 nm wavelength range (c=0.0001 g/cm^3; μ=4.73*10^{12} Da/cm, L=5 μm; the dashed lines represent the best linear fit to each dataset)

Effect of Fiber Length:

All three of the methods evaluated to extract diameter and mass-length ratio from turbidimetry values assume that the fibers are infinitely long, but in realistic scenarios fiber lengths range from 0.3-4.8 μm [1]. Figure S.9 shows the representative plots of $\frac{\left(\frac{dn}{dc}(\lambda)\right)^2}{<\frac{dn}{dc}>^2}\frac{n(\lambda)}{<n>}\frac{c}{\tau\lambda^3}$ vs. $\frac{n^2(\lambda)}{<n>^2}\frac{1}{\lambda^2}$ and $\frac{\tau\lambda^5}{A'}$ vs. $\frac{\lambda^2}{n(\lambda)^2}$ for fibers of 100 nm diameter and 4.73*10^{12} Da/cm mass-length ratio created using full light scattering theory (Equations 13-14) at lengths ranging from 0.5-10 μm. As can be seen, the data from the full light scattering theory are less linear with decreasing fiber lengths. Since the three approaches apply a linear fit to the data, this nonlinearity in the full light scattering data results in some inaccuracies in the values determined by the approaches, as described in the discussion section of the main text.

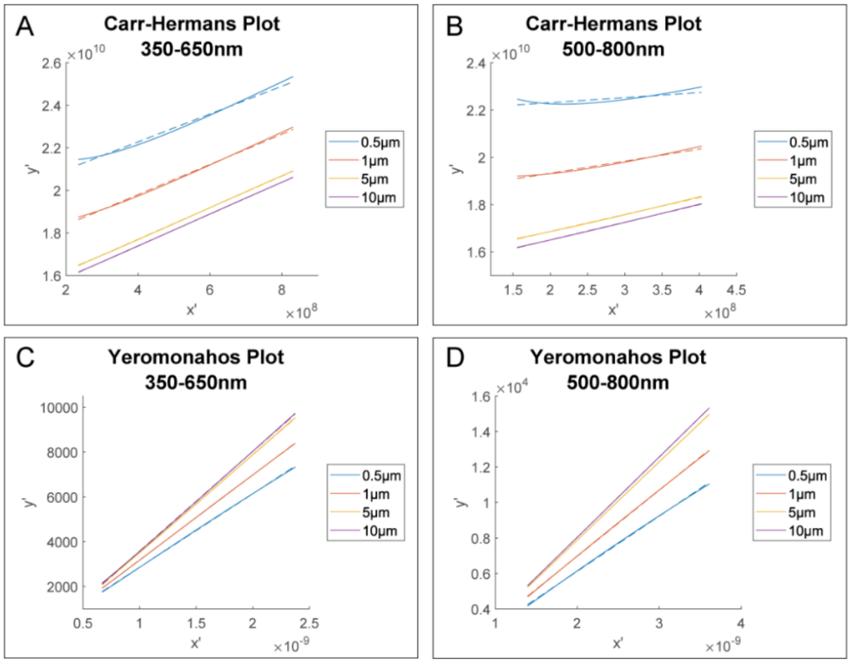

Fig. S.9: Theoretical turbidity values created using full light scattering theory for lengths of 0.5, 1, 5, and 10 μm are plotted in A) the Carr-Hermans format of $\frac{\left(\frac{dn}{dc}(\lambda)\right)^2}{<\frac{dn}{dc}>^2}\frac{n(\lambda)}{<n>}\frac{c}{\tau\lambda^3}$ vs. $\frac{n^2(\lambda)}{<n>^2}\frac{1}{\lambda^2}$ for 350-650 nm and B) 500-800 nm and C) the Yeromonahos format of $\frac{\tau\lambda^5}{A'}$ vs. $\frac{\lambda^2}{n(\lambda)^2}$ for 350-650 nm and D) 500-800 nm. (d=100 nm, μ=4.73*10^{12} Da/cm, c=0.0001 g/cm^3; the dashed lines represent the best linear fit to each dataset)

Effect of Concentration:

A theoretical turbidity dataset was created using the full light scattering theory (Equations 13-14) for concentrations of 0.001 g/cm^3, 0.008 g/cm^3, 0.0001 g/cm^3, and 0.00001 g/cm^3 (physiological values are between 0.0015 and 0.004 g/cm^3). There was no difference in the percent error between the values calculated using the three approaches compared to the values used to create the full light scattering theory dataset with changing fibrinogen concentration. Therefore, the percent error is independent of fibrinogen concentration. This is due to the fact that the Rayleigh ratio, R(θ), depends linearly on concentration, and therefore turbidity depends linearly on the concentration. Then since for the Carr-Hermans approach (corrected for the wavelength dependence of n and dn/dc) the y-data in the plots contains c in the numerator and turbidity in the denominator ($\frac{\left(\frac{dn}{dc}(\lambda)\right)^2}{<\frac{dn}{dc}>^2}\frac{n(\lambda)}{<n>}\frac{c}{\tau\lambda^3}$), the concentration dependence will cancel out, resulting in the full light scattering data being independent of fibrinogen concentration. Similarly, for the Yeromonahos approaches (corrected for the wavelength dependence of n and dn/dc), turbidity is in the numerator and concentration is in the denominator for the y-data in the plots ($\frac{\tau\lambda^5}{\left(\frac{88}{15}\right)\pi^3 n(\lambda)^3\frac{c}{N_A}\left(\frac{dn}{dc}(\lambda)\right)^2}$), so since turbidity is linearly dependent on concentration, the concentration dependence will cancel out. Therefore, the datasets do not change with changing fibrinogen concentration, causing the percent error in the different approaches to also be independent of fibrinogen concentration.

Effect of Mass-Length Ratio:

A theoretical turbidity dataset was created using the full light scattering theory (Equations 13-14) for mass-length ratios of $2*10^{11}$ Da/cm, $4.73*10^{11}$ Da/cm, $4.73*10^{12}$ Da/cm, and $4.73*10^{13}$ Da/cm. There was no difference in the percent error between the values calculated using the three approaches compared to the values used to create the full light scattering theory dataset with changing mass-length ratio. Therefore, the percent error is independent of mass-length ratio. This is due to the fact that changing the mass-length ratio changes the Rayleigh ratio for full light scattering theory, which in turn changes the turbidity values. This results in a change to the y-data for full light scattering theory plotted in the formats for analysis ($\frac{\left(\frac{dn}{dc}(\lambda)\right)^2}{<\frac{dn}{dc}>^2}\frac{n(\lambda)}{<n>}\frac{c}{\tau\lambda^3}$ for the Carr-Hermans approach with n and dn/dc corrected for wavelength dependence and $\frac{\tau\lambda^5}{\left(\frac{88}{15}\right)\pi^3 n(\lambda)^3\frac{c}{N_A}\left(\frac{dn}{dc}(\lambda)\right)^2}$ for the Yeromonahos approaches with n and dn/dc corrected for wavelength dependence). Therefore, a change in mass-length ratio would cause a difference in the slope and y-intercept of the plots, but it does not change the overall shape of the dataset, and therefore does not impact the fit of a linear line to the data. Thus, the actual values calculated for the diameter and mass-length ratio will change with changing mass-length ratio, but the percent error in the calculations would be unaffected.

S.7 Larger Diameter Values

Figure S.10 and S.11 below show the percent error in the diameter and mass-length ratio, respectively, but for diameters ranging from 20-400 nm, instead of for 10-200 nm as Figures 5 and 6 in the main text of this paper do. The error in all three turbidimetry approaches increases for both the estimates of the diameter and the mass-length ratio above diameters of 200 nm. This is the case for both the 350-650 nm wavelength range as well as the 500-800 nm wavelength range. As mentioned in the discussion section of the main text, the full light scattering theory (Equations 13 and 14) uses a form factor for “thin” rods, suggesting that as diameters increase and approach the wavelength, the form factor equation becomes less applicable. This may explain why the percent error increases above diameters of 200 nm.

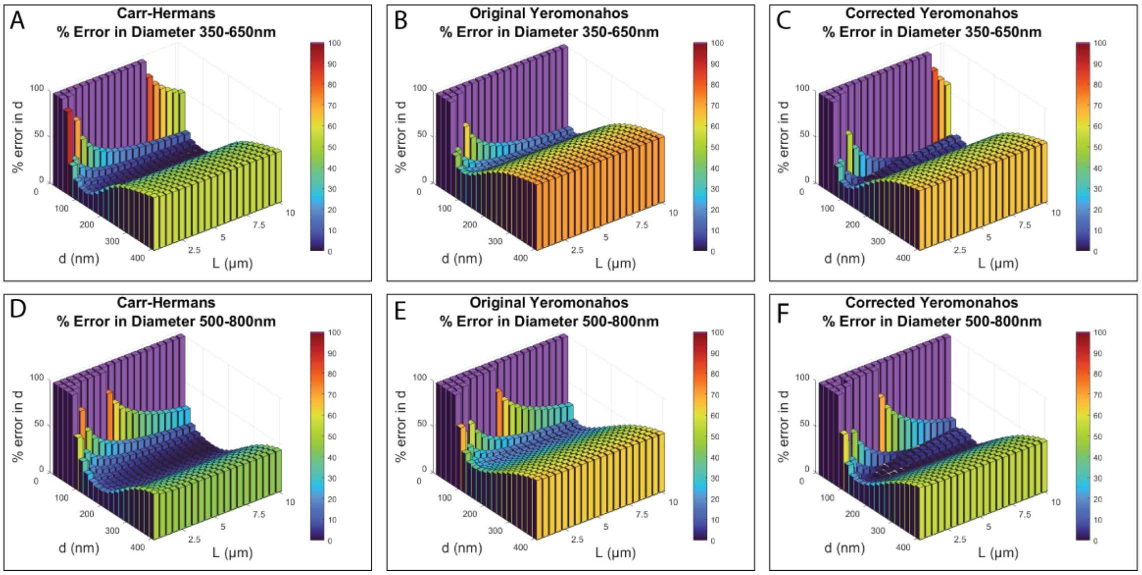

Fig. S.10: Percent error between the diameter obtained from fitting the three approaches to theoretical turbidity values created using full light scattering theory and the value used to create the initial dataset for lengths of 0.5-10 μm and diameters of 20-400 nm for wavelength ranges of 350-650 nm (A-C) and 500-800 nm (D-F). (c=0.0001 g/cm^3, μ=4.73*10^{12} Da/cm, dn/dc and n corrected for wavelength dependence for fibers in 150 mM sodium chloride, 20 mM HEPES, pH 7.4; the purple bars represent imaginary diameter values calculated from the fit and were assigned a value of 100% error)

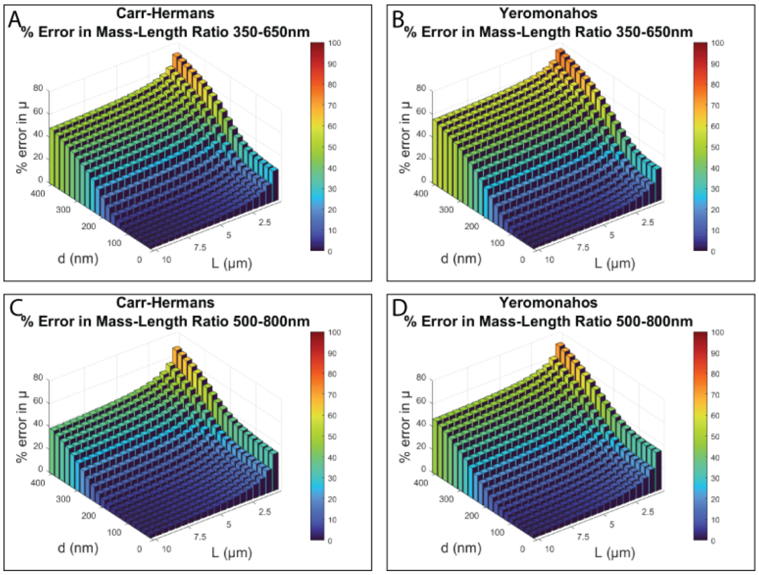

Fig. S.11: Percent error between the mass-length ratio obtained from fitting the approaches to theoretical turbidity values created using full light scattering theory and the value used to create the initial dataset for lengths of 0.5-10 μm and diameters of 20-400 nm for wavelength ranges of 350-650 nm (A-B) and 500-800 nm (C-D). (c=0.0001 g/cm^3, μ=4.73*10^{12} Da/cm, dn/dc and n corrected for wavelength dependence for fibers in 150 mM sodium chloride, 20 mM HEPES, pH 7.4)

S.8 Experimental Turbidimetry Analysis

The three investigated turbidimetric approaches were fit to experimental data for a fibrin clot formed from either 0.5 mg/mL fibrinogen and 0.1 NIH-U/mL thrombin, or 1 mg/mL fibrinogen, 0.1 NIH-U/mL thrombin, and 25 L-U/mL FXIIIa, in a buffer of 150 mM sodium chloride, 20 mM HEPES, 5 mM calcium chloride, pH 7.4. Since this buffer contains calcium chloride, the equations accounting for the wavelength dependence of n and dn/dc differ slightly from the values given in the main text for HBS buffer. The equations become:

$$n(\lambda) = 1.3247 + \frac{3093.9}{\lambda^2} \quad [S.19]$$

$$\frac{dn}{dc}(\lambda) = 0.1853 + \frac{1689.9}{\lambda^2} \quad [S.20]$$

where λ is the wavelength in nanometers. These equations were obtained using the software program SEDNTERP [6] and SEDFIT [7,8], respectively. When using constant values of n and dn/dc, the spectral averages were used, which are <n> = 1.3383 and <dn/dc> = 0.192728 for a wavelength range of 350-650 nm and <n> = 1.33243 and <dn/dc> = 0.189525 for a wavelength range of 500-800 nm.

The plots of the experimental data from 0.5 mg/mL fibrinogen can be seen in Figure S.12 and Figure S.13 for the wavelength dependence of n and dn/dc being accounted for, and for constant values of n and dn/dc being used, respectively, and in Figure S.14 and S.15, respectively, for the clot from 1 mg/mL fibrinogen. The Carr-Hermans plots, particularly in the wavelength range of 350-650 nm show an increased curvature in the data, which is particularly present when using a constant value for n and dn/dc. This matches the expected results based on theory as seen in Figure S.4 for larger diameter values, except that the curvature is in the opposite direction. The values of diameter obtained from fitting the turbidimetric approaches to the datasets are shown in Table 2 of the main text for both clot conditions.

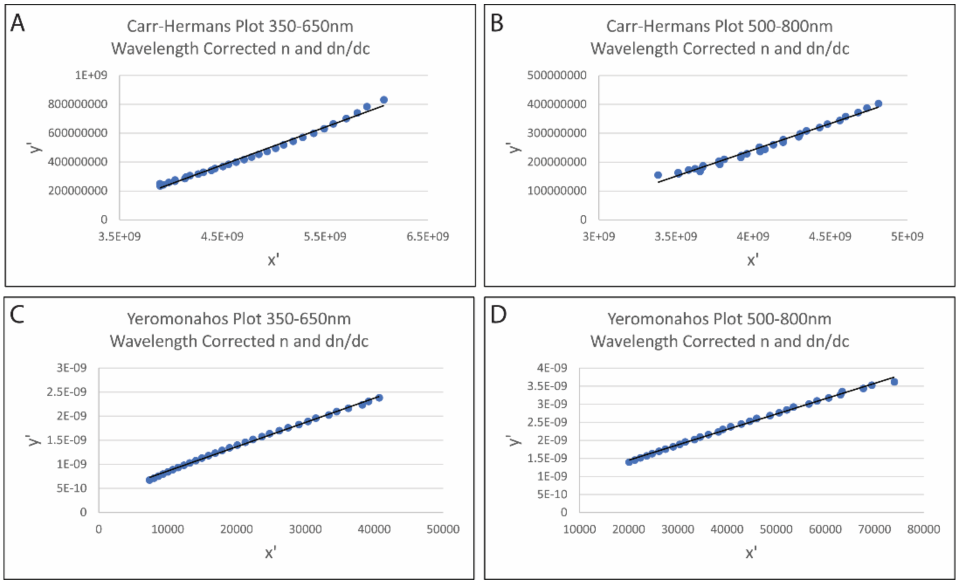

Fig. S.12: Wavelength corrected experimental turbidimetry data from a clot formed from 0.5 mg/mL fibrinogen and 0.1 NIH-U/mL thrombin (in HBS buffer + 5 mM calcium chloride) plotted in A) the Carr-Hermans format of $\frac{\left(\frac{dn}{dc}(\lambda)\right)^2}{<\frac{dn}{dc}>^2}\frac{n(\lambda)}{<n>}\frac{c}{\tau\lambda^3}$ vs. $\frac{n^2(\lambda)}{<n>^2}\frac{1}{\lambda^2}$ for 350-650 nm and B) 500-800 nm and C) the Yeromonahos format of $\frac{\tau\lambda^5}{A'}$ vs. $\frac{\lambda^2}{n(\lambda)^2}$ for 350-650 nm and D) 500-800 nm. (the solid lines represent the best linear fit to each dataset)

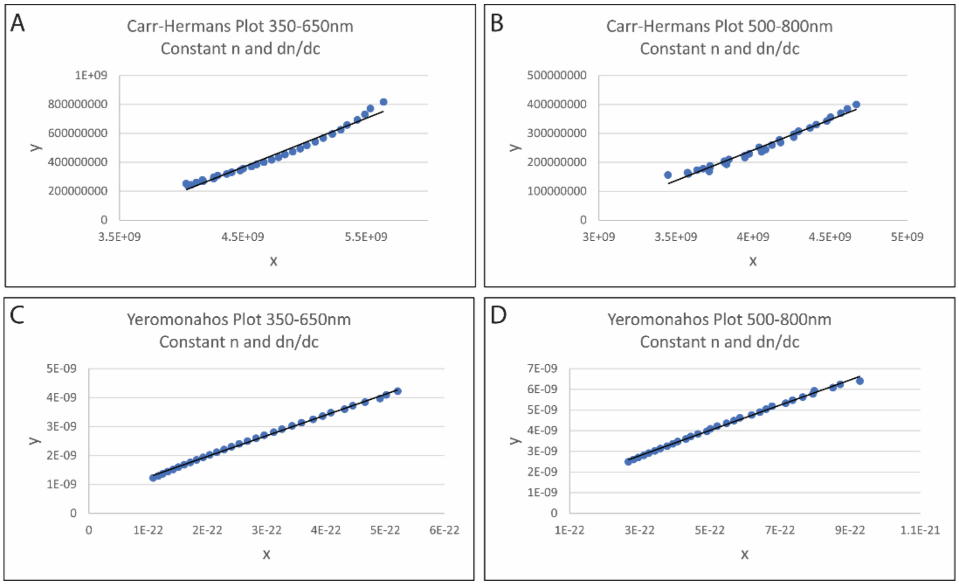

Fig. S.13: Experimental turbidimetry data from a clot formed from 0.5 mg/mL fibrinogen and 0.1 NIH-U/mL thrombin (in HBS buffer + 5 mM calcium chloride) plotted in A) the Carr-Hermans format of $\frac{c}{\tau\lambda^3}$ vs. $\frac{1}{\lambda^2}$ for 350-650 nm and B) 500-800 nm and C) the Yeromonahos format of $\tau\lambda^5$ vs. λ^2 for 350-650 nm and D) 500-800 nm. (the solid lines represent the best linear fit to each dataset)

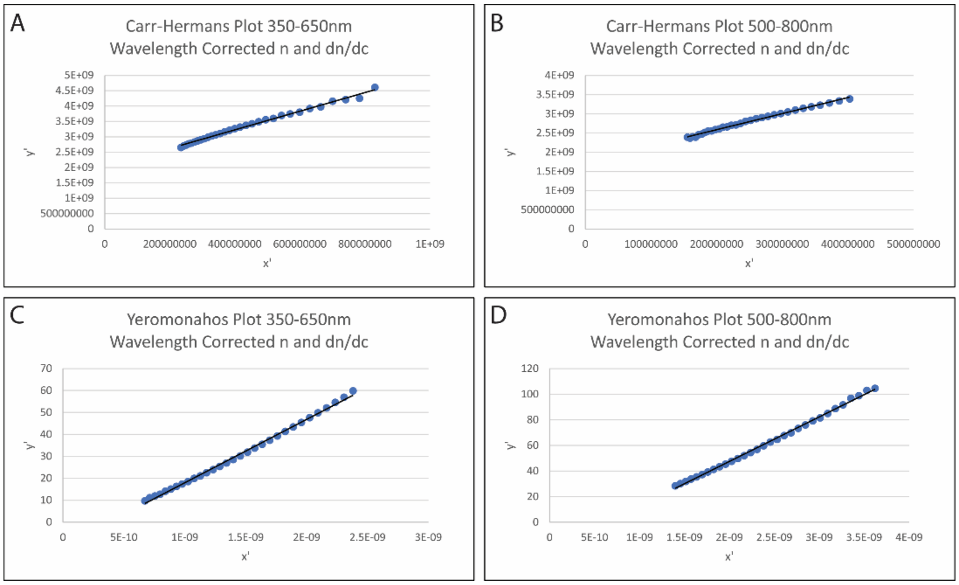

Fig. S.14: Wavelength corrected experimental turbidimetry data from a clot formed from 1 mg/mL fibrinogen, 0.1 NIH-U/mL thrombin, 25 L-U/mL FXIIIa (in HBS buffer + 5 mM calcium chloride) plotted in A) the Carr-Hermans format of $\frac{\left(\frac{dn}{dc}(\lambda)\right)^2}{<\frac{dn}{dc}>^2}\frac{n(\lambda)}{<n>}\frac{c}{\tau\lambda^3}$ vs. $\frac{n^2(\lambda)}{<n>^2}\frac{1}{\lambda^2}$ for 350-650 nm and B) 500-800 nm and C) the Yeromonahos format of $\frac{\tau\lambda^5}{A'}$ vs. $\frac{\lambda^2}{n(\lambda)^2}$ for 350-650 nm and D) 500-800 nm (the solid lines represent the best linear fit to each dataset)

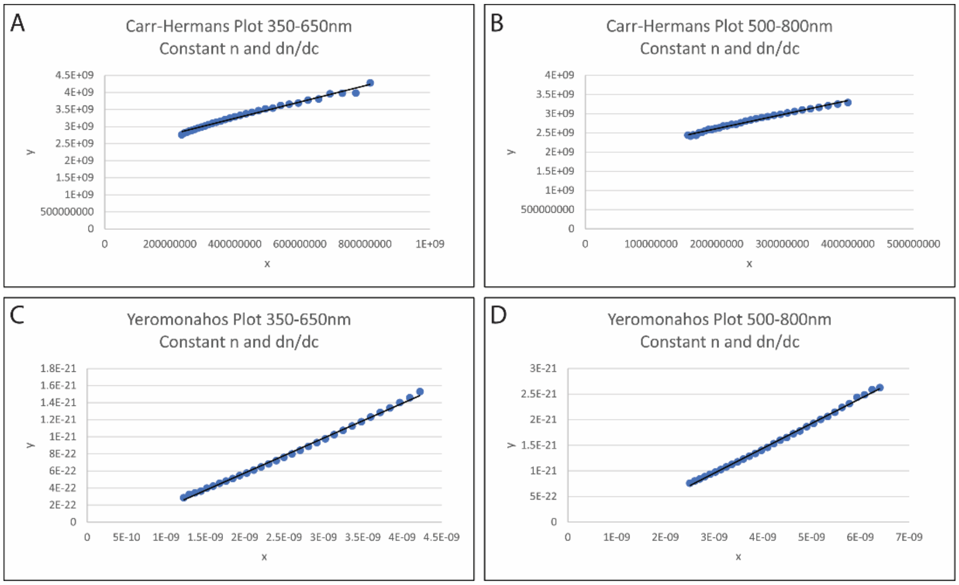

Fig. S.15: Experimental turbidimetry data from a clot formed from 1 mg/mL fibrinogen, 0.1 NIH-U/mL thrombin, 25 L-U/mL FXIIIa (in HBS buffer + 5 mM calcium chloride) plotted in A) the Carr-Hermans format of $\frac{c}{\tau\lambda^3}$ vs. $\frac{1}{\lambda^2}$ for 350-650 nm and B) 500-800 nm and C) the Yeromonahos format of $\tau\lambda^5$ vs. λ^2 for 350-650 nm and D) 500-800 nm (the solid lines represent the best linear fit to each dataset)

For the Carr-Hermans approach accounting for the wavelength dependence of n and dn/dc, the diameter value was obtained using the equation:

$$d = \sqrt{\frac{77}{23}\frac{1}{<n>^2\pi^2}\frac{slope}{yintercept}} \qquad [S.21]$$

and the uncertainty in the diameter was obtained using the equation:

$$\delta d = \sqrt{\begin{array}{c}\left(\frac{1}{2}\left(\frac{77}{23}\frac{1}{<n>^2\pi^2}\right)^{\frac{1}{2}}\left(\frac{slope}{yintercept}\right)^{-\frac{1}{2}}\frac{1}{yintercept}\right)^2\delta slope^2 \\ +\left(-\frac{1}{2}\left(\frac{77}{23}\frac{1}{<n>^2\pi^2}\right)^{\frac{1}{2}}(slope)^{\frac{1}{2}}(yintercept)^{-\frac{3}{2}}\right)^2\delta yintercept^2\end{array}} \qquad [S.22]$$

For the Carr-Hermans approach using constant values of n and dn/dc, the diameter value was obtained using the equation:

$$d = \sqrt{\frac{77}{23}\frac{1}{n^2\pi^2}\frac{slope}{yintercept}} \qquad [S.23]$$

and the uncertainty in the diameter was obtained using the equation:

$$\delta d = \sqrt{\begin{array}{c}\left(\frac{1}{2}\left(\frac{77}{23}\frac{1}{n^2\pi^2}\right)^{\frac{1}{2}}\left(\frac{slope}{yintercept}\right)^{-\frac{1}{2}}\frac{1}{yintercept}\right)^2\delta slope^2 \\ +\left(-\frac{1}{2}\left(\frac{77}{23}\frac{1}{n^2\pi^2}\right)^{\frac{1}{2}}(slope)^{\frac{1}{2}}(yintercept)^{-\frac{3}{2}}\right)^2\delta yintercept^2\end{array}} \qquad [S.24]$$

For the Yeromonahos approaches accounting for the wavelength dependence of n and dn/dc, the diameter value was obtained using the equation:

$$d = 2\sqrt{\frac{-yintercept}{(slope)B\pi^2}} \qquad [S.25]$$

and the uncertainty in the diameter was obtained using the equation:

$$\delta d = \sqrt{\begin{array}{c}\left(-\left(\frac{1}{B\pi^2}\right)^{\frac{1}{2}}(-yintercept)^{\frac{1}{2}}(slope)^{-\frac{3}{2}}\right)^2\delta slope^2 \\ +\left(-\left(\frac{1}{B\pi^2}\right)^{\frac{1}{2}}(slope)^{-\frac{1}{2}}(-yintercept)^{-\frac{1}{2}}\right)^2\delta yintercept^2\end{array}} \qquad [S.26]$$

where B = 184/154 for the original Yeromonahos approach and 184/231 for the corrected Yeromonahos approach.

For the Yeromonahos approaches using constant values of n and dn/dc, the diameter value was obtained using the equation:

$$d = 2\sqrt{\frac{-yintercept}{(slope)B\pi^2 n^2}} \qquad [S.27]$$

and the uncertainty in the diameter was obtained using the equation:

$$\delta d = \sqrt{\begin{array}{c}\left(-\left(\frac{1}{B\pi^2 n^2}\right)^{\frac{1}{2}}(-yintercept)^{\frac{1}{2}}(slope)^{-\frac{3}{2}}\right)^2\delta slope^2 \\ +\left(-\left(\frac{1}{B\pi^2 n^2}\right)^{\frac{1}{2}}(slope)^{-\frac{1}{2}}(-yintercept)^{-\frac{1}{2}}\right)^2\delta yintercept^2\end{array}} \qquad [S.28]$$

with B being the same as for the equations accounting for the wavelength dependence of n and dn/dc described above.

S.9 STORM Imaging

In addition to fitting turbidimetry data with each of the investigated approaches to determine the fiber diameter, the diameter for clots of these fibrinogen and thrombin concentrations were also determined using stochastic optical reconstruction microscopy (STORM) imaging, which provides images with a resolution of approximately 20 nm, allowing it to be used to accurately determine fiber diameters above that length.

Two different clots at a concentration of 0.5 mg/mL fibrinogen and 0.1 NIH-U/mL thrombin were imaged using STORM microscopy, with a total of 77 fiber diameters measured. A histogram of the acquired diameters is shown in Figure S.16. The first sample yielded an average diameter of 182 ± 36 nm, and the second sample yielded an average fiber diameter of 179 ± 38 nm. Thus both samples gave similar results and we combined the data to get a final mean value.

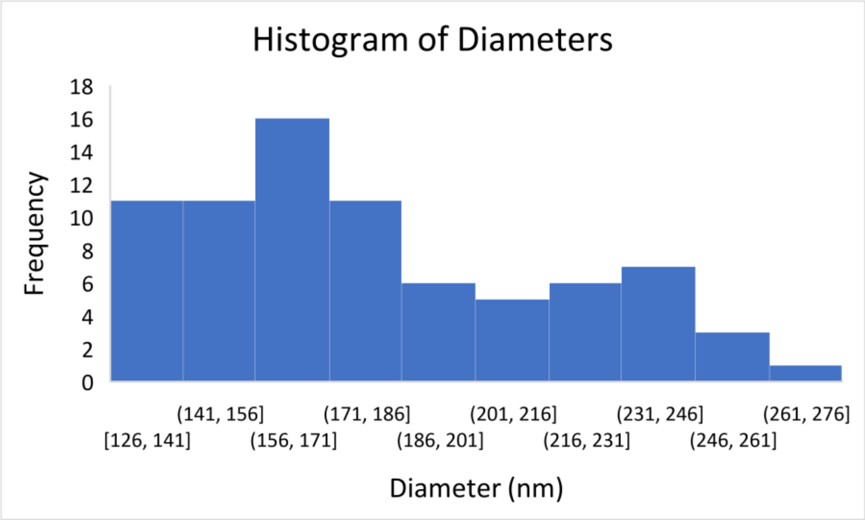

Fig. S.16: Histogram of diameter values obtained from stochastic optical reconstruction microscopy (STORM) imaging of a clot formed from 0.5 mg/mL fibrinogen and 0.1 NIH-U/mL thrombin (in HBS buffer + 5 mM calcium chloride) obtained on a Nikon Ti2-E inverted microscope using a 100X oil objective (diameter values measured using ImageJ).

A sample formed from 1 mg/mL fibrinogen, 0.1 NIH-U/mL thrombin, and 25 L-U/mL was also imaged using STORM microscopy, with a total of 128 fiber diameters measured. A histogram of the acquired diameters is shown in Figure S.17.

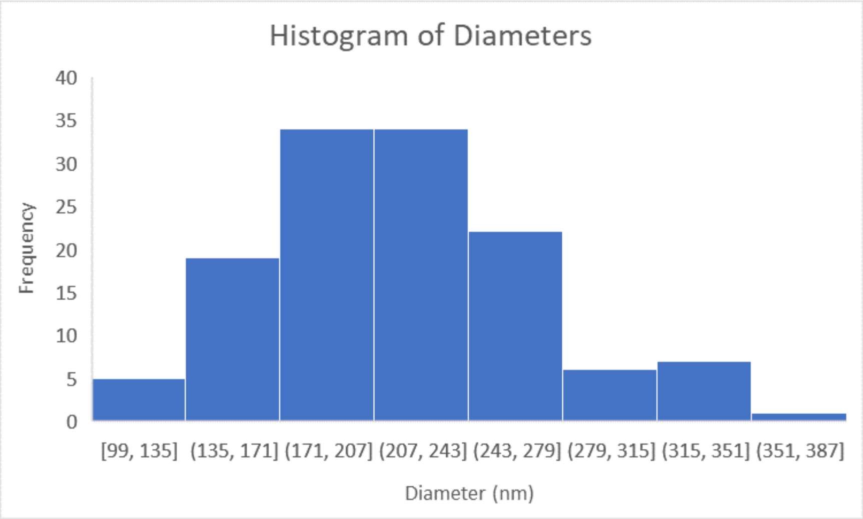

Fig. S.17: Histogram of diameter values obtained from stochastic optical reconstruction microscopy (STORM) imaging of a clot formed from 1 mg/mL fibrinogen, 0.1 NIH-U/mL thrombin, 25 L-U/mL FXIIIa (in HBS buffer + 5 mM calcium chloride) obtained on a Nikon Ti2-E inverted microscope using a 100X oil objective (diameter values measured using ImageJ)

The percent error between the diameter value obtained from experimental data using the turbidimetry approach to that obtained using STORM imaging in comparison to the expected percent error according to the numerical simulations can be seen in Table 4 of the main text for the clot containing 0.5 mg/mL fibrinogen, and in Table S.3 below for the clot containing 1 mg/mL fibrinogen. A discussion of these results is provided in the main text

A

**Wavelength Corrected n and dn/dc**		
	Experimental Percent Error	Predicted Percent Error
Carr-Hermans 350-650 nm	22%	28%
Carr-Hermans 500-800 nm	2%	10%
Original Yeromonahos 350-650 nm	48%	50%
Original Yeromonahos 500-800 nm	33%	36%
Corrected Yeromonahos 350-650 nm	36%	38%
Corrected Yeromonahos 500-800 nm	17%	22%

B

**Constant n and dn/dc**		
	Experimental Percent Error	Predicted Percent Error
Carr-Hermans 350-650 nm	35%	40%
Carr-Hermans 500-800 nm	12%	19%
Original Yeromonahos 350-650 nm	52%	53%
Original Yeromonahos 500-800 nm	36%	39%
Corrected Yeromonahos 350-650 nm	41%	43%
Corrected Yeromonahos 500-800 nm	21%	26%

Table S.3: The percent error in the diameter values obtained experimentally using the turbidimetric fitting approaches (middle column) and the percent error predicted by the numerical simulations (right column) with A) the wavelength dependence of n and dn/dc accounted for and B) using constant values of n and dn/dc.

Supporting References

1. Ryan, E. A., L. F. Mockros, J. W. Weisel, L. Lorand. 1999. Structural Origins of Fibrin Clot Rheology. Biophys. J. 77:2813-2826. doi:10.1016/S0006-3495(99)77113-4.
2. Ferri, F., G.R. Calegari, M. Molteni, B. Cardinali, D. Magatti, M. Rocco. 2015. Size and Density of Fibers in Fibrin and Other Filamentous Networks from Turbidimetry: Beyond a Revisited Carr-Hermans Method, Accounting for Fractality and Porosity. Macromolecules. 48:5423-5432. doi:10.1021/acs.macromol.5b00893.
3. Yeromonahos, C. 2011. Nanostructure des fibres de fibrine. Université de Grenoble. HAL Id:tel-00639435.
4. Carr, M. E., and J. Hermans. 1978. Size and Density of Fibrin Fibers from Turbidity. Macromolecules. 11:46-50. doi:10.1021/ma60061a009.
5. García, X., L. Seyve, Z. Tellier, G. Chevreux, N. Bihoreau, B. Polack, F. Caton. 2020. Aggregates Dramatically Alter Fibrin Ultrastructure. Biophys. J. 117:172-181. doi:10.1016/j.bpj.2019.10.034.
6. Philo, J. SEDNTERP. 2021. Version 3.0.3.
7. Schuck, P. SEDFIT. 2018. Version 16.1c. National Institutes of Health.
8. Schuck, P. 2000. Size-Distribution Analysis of Macromolecules by Sedimentation Velocity Ultracentrifugation and Lamm Equation Modeling. Biophys. J. 78:1606-1619. doi:10.1016/S0006-3495(00)76713-0.
